# PCBP1 depletion promotes tumorigenesis through attenuation of p27^Kip1^ mRNA stability and translation

**DOI:** 10.1186/s13046-018-0840-1

**Published:** 2018-08-07

**Authors:** Hongshun Shi, Hui Li, Ronghua Yuan, Wen Guan, Xiaomei Zhang, Shaoyang Zhang, Wenliang Zhang, Fang Tong, Li Li, Zhihong Song, Changwei Wang, Shulan Yang, Haihe Wang

**Affiliations:** 1 Centre for Translational Medicine, the First Affiliated Hospital, 58 Second Zhongshan Road, Guangzhou, 510080 China; 2 Department of Biochemistry, Zhongshan School of Medicine, 74 Second Zhongshan Road, Guangzhou, 510080 China; 3 Center for Stem Cell Biology and Tissue Engineering, Key laboratory of ministry of education, Sun Yat-sen University, 74 Second Zhongshan Road, Guangzhou, 510080 China; 4 Department of General Surgery, The Second Affiliated Hospital of Nantong University, Nantong University, Nantong, 226001 China; 5 Guangzhou Institutes of Biomedicine and Health, Chinese Academy of Sciences, Guangzhou, 510530 China

**Keywords:** PCBP1, p27, mRNA stability, Carcinogenesis

## Abstract

**Background:**

Poly C Binding Protein 1 (PCBP1) is an RNA-binding protein that binds and regulates translational activity of subsets of cellular mRNAs. Depletion of PCBP1 is implicated in various carcinomas, but the underlying mechanism in tumorigenesis remains elusive.

**Methods:**

We performed a transcriptome-wide screen to identify novel bounding mRNA of PCBP1. The bind regions between PCBP1 with target mRNA were investigated by using point mutation and luciferase assay. Cell proliferation, cell cycle, tumorigenesis and cell apoptosis were also evaluated in ovary and colon cancer cell lines. The mechanism that PCBP1 affects p27 was analyzed by mRNA stability and ribosome profiling assays. We analyzed PCBP1 and p27 expression in ovary, colon and renal tumor samples and adjacent non-tumor tissues using RT-PCR, Western Blotting and immunohistochemistry. The prognostic significance of PCBP1 and p27 also analyzed using online databases.

**Results:**

We identified cell cycle inhibitor p27^Kip1^ (p27) as a novel PCBP1-bound transcript. We then demonstrated that binding of PCBP1 to p27 3’UTR via its KH1 domain mainly stabilizes p27 mRNA, while enhances its translation to fuel p27 expression, prior to p27 protein degradation. The upregulated p27 consequently inhibits cell proliferation, cell cycle progression and tumorigenesis, whereas promotes cell apoptosis under paclitaxel treatment. Conversely, knockdown of PCBP1 in turn compromises p27 mRNA stability, leading to lower p27 level and tumorigenesis in vivo. Moreover, forced depletion of p27 counteracts the tumor suppressive ability of PCBP1 in the same PCBP1 over-expressing cells. Physiologically, we showed that decreases of both p27 mRNA and its protein expressions are well correlated to PCBP1 depletion in ovary, colon and renal tumor samples, independent of the p27 ubiquitin ligase Skp2 level. Correlation of PCBP1 with p27 is also found in the tamoxifen, doxorubincin and lapatinib resistant breast cancer cells of GEO database.

**Conclusion:**

Our results thereby indicate that loss of PCBP1 expression firstly attenuates p27 expression at post-transcriptional level, and subsequently promotes carcinogenesis. PCBP1 could be used as a diagnostic marker to cancer patients.

**Electronic supplementary material:**

The online version of this article (10.1186/s13046-018-0840-1) contains supplementary material, which is available to authorized users.

## Background

Poly(rC)-binding protein 1 (PCBP1) belongs to the heterogeneous nuclear ribonucleoprotein (hnRNP) family which is composed of hnRNP K/J and the alpha-complex proteins (PCBP1–4α or CP1–4) containing three hnRNP K homology (KH) domains for RNA-binding [1]. Thus, PCBP1 and PCBP2 are also known as hnRNP E1 and hnRNP E2 [2, 3], respectively, and share 89% of amino acid similarity [4].

PCBP1 is ubiquitously expressed and functionally plays multiple roles in transcriptional and posttranscriptional regulations, including mRNA stabilization [5, 6]. PCBP1 regulates gene expression via binding to specific elements of target mRNAs with AU-rich elements (AREs) or U-rich elements located in 3’-untranslated regions (3’-UTR) (e.g. *AR, p21, p63, eNOS*) [7–10] or in 5’-UTRs (e.g. *c-myc, PRL-3, EV71*) [6, 11, 12]. PCBP1 also inhibits the alternative spicing of CD44 [13] or its alternative polyadenylation [14]. Recent studies indicate that PCBP1 phosphorylation at Ser43 by Akt2 upon TGF-beta stimulation promotes epithelial to mesenchymal transition in various cancer cells [15–19]. PCBP1 also functions as transport protein with PCBP2 together to deliver irons to ferritin for storage, or binds to prolyl and asparagyl hydroxylases to metalate the mononuclear iron center [20–22]. In addition, PCBP1 modulates housekeeping degradation of mitochondrial antiviral signaling (MAVS) involved in antiviral immunity and anti-inflammation [23]. Moreover, PCBP1 can bind with RACK1 to regulate MOR expression [24].

Increasing evidence indicates that PCBP1 could be involved in repressing carcinogenesis, as downregulation of PCBP1 has been observed in multiple cancers, including cervical cancer [25], liver cancer [13], breast cancer cells [26], colon cancer and lung cancer [6]. Our previous study revealed that PCBP1 translationally represses metastatic PRL-3 and its overexpression inhibits tumorigenesis, whereas PCBP1 knockdown in turn enhances tumor formation. However, the simultaneous inverse correlation of PCBP1 protein level to that of PRL-3 is observed in only 37% lung and 24% colon carcinoma samples, as PCBP1 silence fails to provoke PRL-3 upregulation in some tumor samples [6], indicating that PCBP1 could play multiple roles in tumor suppression, rather than only by delaying PRL-3 translation. So far, the underlying mechanism of PCBP1 in tumor suppression remains elusive.

Generally, protein level is not only determined by mRNA amount, but the posttranscriptional regulation. To thoroughly investigate PCBP1’s anti-tumorigenic character, here we first applied the transcriptome-wide RNA immunoprecipitation (RIP) to identify the stably bound mRNAs to PCBP1, and followed by high-throughout RNA sequencing. Of note, PCBP1 was bound to a class of mRNAs, in which cell cycle inhibitor p27^Kip1^ (p27) was notably identified as a novel PCBP1-bound transcript. We characterized that binding of PCBP1 to p27 mRNA 3’-UTR stabilized p27 mRNA, consequently increased p27 protein expression to induce cell cycle arrest, inhibit cell proliferation, and repress tumorigenesis both in vitro and in vivo. Eventually, we also demonstrated that loss of PCBP1 mRNA and protein were positively related to p27 downregulation in clinical tumor samples, indicating the general importance of p27 loss in tumorigenesis.

## Methods

### Cell culture

A2780 cells were cultured in DMEM (Hyclone), MDA-MB-231 cells were cultured in high-glucose DMEM, so as DLD-1 cells in RMPI-1640 medium. All media were supplemented with 10% fetal bovine serum (FBS) and 1% penicillin-streptomycin antibiotics. Cells were cultured at 37 °C with 5% CO_2_ in incubator (Thermo).

### Immunoprecipitation and sequencing identification of GFP-PCBP1 bound mRNAs

A2780-GFP and A2780-GFP-PCBP1 cells were grown to 90% of cell confluency and washed with cold PBS, then treated for crosslinking of PCBP1 to the bound transcripts with 1% paraformaldehyde-PBS at 37 °C for 20 min on rocking platform and stopped with 0.2 M glycine-PBS at 37 °C for 5 min, followed by washing another 3 times with ice-cold PBS and harvesting cells by scraping in ice-cold PBS. Harvested cells were centrifuged at 2500 rpm for 5 min and lysed in 500 μl ice-cold hypotonic buffer (10 mM HEPES pH 7.0, 100 mM KCl, 5 mM MgCl_2_, 0.5% NP40 and 1 mM DTT, 1× Protease Inhibitor Cocktail (Roche), 100 units/ml RNase inhibitors) on ice for 5 min to swell, and subsequently aliquoted and frozen at − 80 °C overnight. The lysates were thawed quickly and centrifuged at 14,000 rpm for 10 min at 4 °C and the supernatants were collected and quantified by BCA protein assay using GENios Plus Microplate Reader (Tecan).

For RNA-Protein Immunoprecipitation (RIP), μMACS™ Epitope Tag Protein Isolation Kits (Miltenyi Biotec) were used. Briefly, 2 mg total protein from the above preparations were pre-cleared by incubating with Protein A sepharose (GE Healthcare) and 4 μg normal rabbit IgG (Santa Cruz) in NT2 buffer (50 mM Tris-HCl pH 7.4, 150 mM NaCl, 1 mM MgCl_2_ and 0.05% NP40) with 0.1 μg/ml BSA (Sigma) and 0.1 μg/ml yeast tRNA (Ambion) at 4 °C for 1 h on shaker. After centrifuging, the supernatant were incubated with 50 μl Anti-GFP MicroBeads (Miltenyi Biotec), 0.5 mg/ml yeast tRNA and 0.5 mg/ml BSA at 4 °C for 1 h with rotation. Bound protein-RNA complexes were enriched and the bound RNAs were then extracted with elution buffer containing 6 μl 5 M NaCl containing 20 μg proteinase K in 150 μl volume at 42 °C for 1 h, and 65 °C for 1 h more, followed by phenol:chloroform clearance and Na-acetate:lithium chloride precipitation. The mRNAs on the purified RNAs were amplified with TransPlex® Complete Whole Transcriptome Amplification Kit (Sigma) according to the manufacturer’s instruction for sequencing. RNA-sequencing was conducted by Ribo Bio Co., Ltd., China and the enriched mRNAs were confirmed by RT-PCR analysis.

### Semi-quantitative and quantitative RT-PCR analyses

Total RNA was isolated from cells or clinical tumor samples with Ultrapure RNA kit (CWBiotech) and the complementary DNA (cDNA) was synthesized by using Transcriptor First Strand cDNA Synthesis System kit (Roche) with Oligo (dT)_18_. For semi-quantitative analysis, PCR amplification was performed with GoTaq® DNA polymerase (Promega), 1 μM of each pair of the indicated primers (Additional file 1: Table S1), and 0.5 μl cDNA for a 2 min initial denaturation at 95 °C, followed by 20–27 cycles of 30 s at 95 °C, 30 s at the appropriate anneal time, and 1 min at 72 °C. Products were run in 1.5% agarose gel and the band intensity was scanned, and normalized based on their corresponding internal GAPDH control as the relative expression level. Real time PCR was performed by using a SYBR Green reaction kit (TIANGEN, China) in CFX 96 real time PCR instrument(BioRad,USA), according to the supplier’s protocol. All experiments were carried out in triplicate and analyzed using the comparative threshold cycle (2^−ΔΔCT^) method, where the ΔΔCT is the difference between normalized target gene and internal control (ΔCT sample – ΔCT control = ΔΔCT).

### Plasmid constructions

To obtaining the 3’-UTR of human p27 mRNA, total RNA was extracted from A2780 cells and cDNA (from 2 μg RNA) was synthesized with SuperScript III and primed with oligo (dT) in accordance with the manufacturer’s instructions (Invitrogen). Primers 5’-GCTCTAGAACAGCTCGAATTAAGAATATGT-3′ with an *Xba* I restriction site and 5’-GGATCCAATAGCTATGGAAGTTTT-3′ with a *Bam*H I site were used to amplify the full length of p27 mRNA 3’-UTR. The full length fragment was double digested with *Xba* I and *Bam*H I and subcloned into pGL3-Basic vector (Promega), which is located downstream of the firefly luciferase. The indicated primers (Additional file 1: Table S2) were used to generate deletions of p27 mRNA 3’-UTR (constructs a-m).

To obtain point mutation of human PCBP1, pEGFP-PCBP1 [6] was used as template to make various point mutations with QuikChange® Site-Directed Mutagenesis Kit (Stratagene), according to the manufacturer’s instruction. Amino acid substitution mutations of G30A, KH2 G114A and G296A in GXXG motif of KH domain were produced to break KH1, KH2 and KH3 of PCBP1, respectively [27]. Similarly, other indicated mutations were also produced to break the phosphorylation sites of PCBP1. All primers for the above mutations are listed in Additional file 1: Table S3.

Two p27 specific shRNA sequences are 5’-CGCAAGTGGAATTTCGACTTT-3' (shRNA1) and 5’-CCCGGTCAATCATGAAGAACT-3’ (shRNA2) [28] which were cloned into pGFP-V-RS shRNA vector (OriGene). All above mentioned constructs were verified by DNA sequencing.

### Cell cycle analysis

A2780 and DLD-1 cells stably expressed PCBP1 KD, GFP-PCBP1, GFP-PCBP1-p27 KD and GFP were seeded and cultured for 24 h, followed with serum-free starvation or combined with 0.08 μM methotrexate (MTX) to synchronize cell phase for 24 h. Cells were cultured for another 12 h or 16 h, then fixed with 70% ethanol in PBS and stained with 25 μg/ml propidium iodide (Beyotime) containing 50 μg/ml RNAse A for 30 min. The treated cells were analyzed with EPICS XL-MCL flow cytometry (Bechman Coulter), and the data were analyzed with FlowJo 7.6 software. In brief, the control samples were loaded first to optimize the conditions and then set the proper parameters to all samples for flowing and analyzing to generate graphical reports.

### Apoptosis analysis

A2780 and DLD-1 cells stably expressed GFP-PCBP1 and GFP were seeded and cultured for 24 h, followed with treatment with 3 μM Paclitaxel for 24-36 h. Cells collected by detaching with 0.25% EDTA-free trypsin and washed with PBS, then stained with APC-conjugated Annexin V and 7-ADD (KeyGEN BioTECH, KGA1017) for 15 min at room temperature in dark. Cells were analyzed by flow cytometry (Beckman, Gallios).

### Transient transfection and luciferase assays

A2780 cells were seeded at a density of 1 × 10^4^ cells/96-well plate one day before and then co-transfected with each p27 mRNA 3’-UTR constructs (a-m) and internal control pRL-TK (Renilla) plasmid with X tremeGENE HP DNA transfect reagent (Roche). Luciferase activities were measured using the Dual-Luciferase-reporter assay system (Promega) at 24 h after transfection with Synergy 2 Muti-Mode Reader (Bio Tek). The relative luciferase activity was normalized based on that of the individually corresponding Renila luciferase, which was considered as the internal control. The presented data are from two independent triplicate experiments, shown as mean ± SD.

### Cell proliferation assay

Cells were seeded 96-well plates with time-course incubations, and were then washed with phosphate-buffered saline (PBS) with MTT (0.5 mg/mL PBS) and incubated at 37 °C for 30 min. Formazan crystals were dissolved with dimethyl sulfoxide (40 μL/well) and detected at OD_570_ using an Emax Endpoint ELISA microplate reader (Molecular Devices).

### Colony formation assay

Colony formation assay was performed using double-layer soft agar in 6-well plates with a bottom layer of 0.5% agar and a top layer of 0.35% agarose. In detail, the 0.5% base agar layer was prepared in 6-well plates first, then the assayed cells were harvested, counted and resuspended in 0.7% top agarose solution, followed by pouring the cell mix on top of the base agar layer and incubating for 10–14 days, while feed cells 2 times a week. At the end of the incubation, cells were fixed with 4% paraformaldehyde-PBS for 5 min and stained with 0.1% crystal violet. Colonies were photographed and counted under stereomicroscope in 5 random fields.

### Tumorigenicity assays in immunodeficient mice

All mouse experiments were conducted under the Institutional Animal Care and Use Committee (IACUC) approved protocols by Sun Yat-sen University. The immunodeficient mice BALB/c-nu were purchased from Guangdong Medical Laboratory Animal Center. Five or six nude mice at 4-week-old in each group were subcutaneously injected with 5 × 10^6^ A2780 cells or 1 × 10^7^ DLD-1 cells overexpressing PCBP1 with additional p27 knockdown, or cells overexpressing PCBP1 alone with GFP control cells into the left and right sides of their armpit area to examine the tumorigenicity of the experimental cells in vivo. After 3 (A2780 cells) or 4 (DLD-1 cells) weeks, the induced tumor sizes were measured and recorded, then the tumors were dissected for weighing. GraphPad Prism 5 and paired t test were used for statistical analysis.

### mRNA stability assay

A2780 cells stably expressed GFP-PCBP1 or GFP control treated with DMSO, 0.5 μg/ml Actinomycin D (Act D) (Biosharp), 20 μM MG132 (Sigma) or combination of Act D with MG132 for 8 h. Total RNA was extracted for cDNA synthesis, and transcript abundances of p27, c-myc mRNAs and controls were detected by semi-quantitative and real time RT-PCR as indicated.

### Measuring the distribution of p27 mRNA by ribosome profiling

For polyribosome isolation [6, 29], cells were incubated with 90 mg/ml cycloheximide (Sigma) for 10 min followed by trypsinization and harvest. Twenty million cells were resuspended in RSB (20 mM Tris-HCl [pH 7.4], 20 mM NaCl, 30 mM MgCl_2_, RNasin, 100 mg/ml Heparin, and 5 mg/ml cycloheximide). An equal volume of lysis buffer (1.2% Triton X-100, 1.2% deoxycholate) was added, followed by incubating on ice for 5 min. The nuclei and cell debris were removed by centrifugation for 3 min at 12,000 rpm. The supernatant was then diluted with an equal volume of dilution buffer (25 mM Tris-HCl [pH 7.4], 25 mM NaCl, 25 mM MgCl_2_, 0.05% Triton X-100, and 500 mg/ml heparin) and 400 ml of the extract was loaded onto 9.5 ml linear 10 to 50% sucrose gradients and centrifuged at 36,000 rpm for 2 h in a SW41 rotor (Beckman). Ten (1 ml each) fractions were collected with the BioComp piston gradient fractionator linked to an EM-1 UV Monitor (BioRad). The fractions were incubated in 1% SDS and proteinase K at 42 °C for 30 min. RNA was purified by Phenol Chloroform extraction followed by ethanol precipitation. p27 mRNA was detected by semi-quantitative RT-PCR and qRT-PCR as mentioned above.

### Immunochemistry (IHC) analyses of PCBP1 and p27 expression in tumor samples

Ovary tissues (18 normal; 21 carcinoma tissues) and 10 pairs of freshly frozen colon tumor tissues as well as 8 pairs of renal tumor samples were collected from Sun Yat-sen University Cancer Center (SYSUCC), under their Standard Experimental Ethics Protocol. Tissues were formalin-fixed, paraffin-embedded and sliced into 6 μm thin sections using Leica BM 2135 microtome, which were subsequently stained with anti-PCBP1 (1:200 dilution, Abcam), anti-p27 (1:200 dilution, ExCell Bio) and Skp2 (1:200 dilution, CST) antibodies. IHC was developed with Polink-1 HRP DAB Detection System (ZSGB-BIO). Each sample was scored semiquantitatively using the immunoreactive score [30] method that is in consideration of the values of immunoreaction intensity and the percentage of tumor cell staining, as described previously [31, 32]. IRS calculation are presented as IRS = SI (staining intensity) × PP (percentage of positive cells), in which SI was determined as 0 = negative; 1 = weak; 2 = moderate; and 3 = strong. Likewise, PP was defined as 0, < 1%; 1, 1–10%; 2, 11–50%; 3, 51–80%; and 4, 80% positive cells. The relative score in Fig. 6b based on IRS are determined as -, 0; +, 1–3; ++, 4–8; +++, 9–12.

### Statistics and GEO data analyses

SPSS 13.0 and Chi-square analysis were used to analyze the relationship between PCBP1 and p27 expression in IHC staining and the statistical significance was defined as *p* < 0.05.

To analysis p27 mRNA expression in ovarian epithelia and the ovary cancer epithelia cells, Gene Expression Omnibus (GEO) dataset GDS3592 was used. Likewise, 3 independent GEO datasets of the drug-resistant subclones of breast cancer cells treated with Tamoxifen (GSE26459), Doxorubincin (GSE24460), and Lapatinib (GSE16179) [33–35] were analyzed to validate the relationship of PCBP1 and p27 mRNA expression in drug resistance, which were presented as the mean ± SD and compared statistically by Student’s t test, using Graphpad Prism (GraphPad Software), n indicates ovary sample number or the drug-resistant breast cancer cell subclones. A *p* value of < 0.05 was considered statistically significant. All supplementary experimental procedures are availble in Additional file 2.

## Results

### PCBP1 binds with p27 mRNA to upregulate p27 expression

**Fig. 1:**
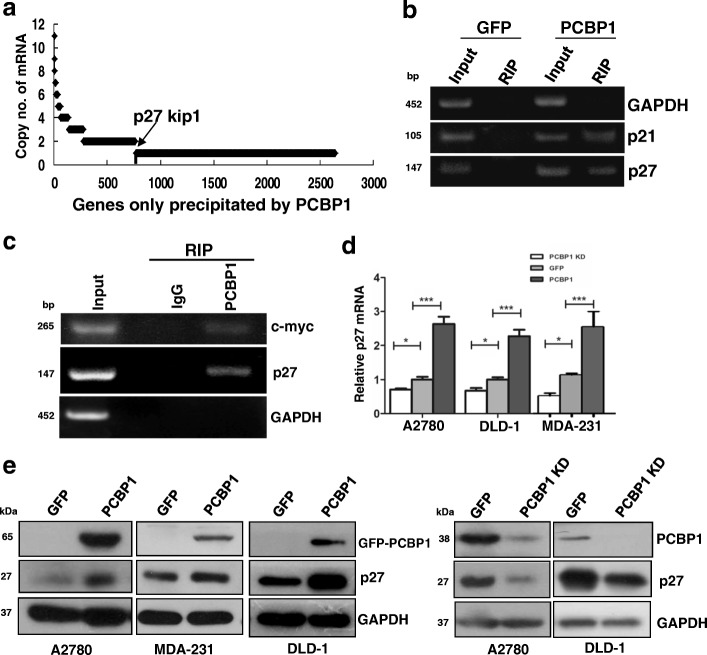
PCBP1 binds to p27 transcript to increase its expression. (**a**) Identification of the purely PCBP1-bound mRNAs by RNA sequencing. mRNA numbers are indicated as copy number. (**b**) RT-PCR validation of PCBP1-associated p27 mRNA from the precipitated mRNA pools in A. p21 and GAPDH transcripts are respectively used as the positive and negative controls of PCBP1-bound mRNA. (**c**) RT-PCR detection of endogenous p27 mRNA immunoprecipitated by PCBP1-specific antibody. Normal IgG is used as negative control for RIP. c-Myc and GAPDH are used as positive or negative control of PCBP1-bound mRNA, respectively. (**d**) Quantitative RT-PCR detection of p27 mRNA in A2780 cells with overexpressing or silencing endogenous PCBP1. Three independent experiments were carried out and analyzed. Data are shown as means±SD. *p<0.05; ***p<0.001. (**e**) Immunoblot of p27 protein expression in the indicated cells with PCBP1 overexpression or silence by its specific shRNAs. A positive correlation between PCBP1 and p27 protein level is observed in these cell lines

Our previous results showed that the frequency of the examined tumor samples with PCBP1 downregulation is much more than that with PRL-3 upregulation [6], implying that PCBP1 could play multiple tumor suppressive roles, instead of just slowing PRL-3 protein translation. To thoroughly investigate the mechanism of PCBP1 against tumor formation, we adopted a strategy to capture all PCBP1-bound mRNAs by crosslinking PCBP1 to RNA molecules and immunoprecipitating them for sequencing identification (indicated as RIP-seq) in human ovarian cancer A2780 cells (Additional file 3: Figure S1A). RNA sequencing showed that p27 is one of the novel mRNAs only bound to PCBP1, but not in the GFP control cells (Fig. 1a and Additional file 1: Table S4). We then specifically focused on whether PCBP1 modulates p27 expression with three reasons: first, cell cycle plays a crucial role in tumorigenesis and cancer progression; second, p27 is one of the most important guardians in cell cycle; third, PCBP1 has been confirmed to interact with p21 mRNA, it is reasonable to inquire the existence of an coordinated interaction between PCBP1 and p27 mRNA to modulate cell cycle.

We sought to verify the association between PCBP1 with p27 mRNA by IP with PCBP1-specific antibody. RT-PCR detection displayed that p27 mRNA was significantly enriched from GFP-PCBP1 cells, but not in the control GFP cells, while p21 mRNA, as a known PCBP1-bound RNA was amplified too, compare to the negative control, GAPDH mRNA which was not detected (Fig. 1b). To further confirm whether endogenous PCBP1 binds to p27 mRNA, we carried out the RIP experiment again and showed that p27 mRNA was really precipitated by PCBP1-specific antibody with a well-known c-myc mRNA bound to PCBP1, but not by normal IgG control (Fig. 1c). Thus, we confirmed the existence of the association between PCBP1 and p27 mRNA in cells. Given the observed association, we speculated that PCBP1 may regulate p27 mRNA level, and the quantitative PCR detection of p27 mRNA in three PCBP1-overexpressing or silencing cell lines indicated that association of PCBP1 to p27 mRNA can increase p27 mRNA level (Fig. 1d). Immunoblots further showed that p27 protein level was increased in the three PCBP1-overexpressing cell lines, whereas p27 downregulation was detected upon PCBP1 knockdown with two specific shRNAs in both A2780 and DLD-1 cells (Fig. 1e), indicating that PCBP1 can upregulate p27 expression through binding to p27 mRNA.

### Binding of PCBP1 to p27 mRNA 3’-UTR favored by PCBP1 phophorylation and its KH1 domain

**Fig. 2:**
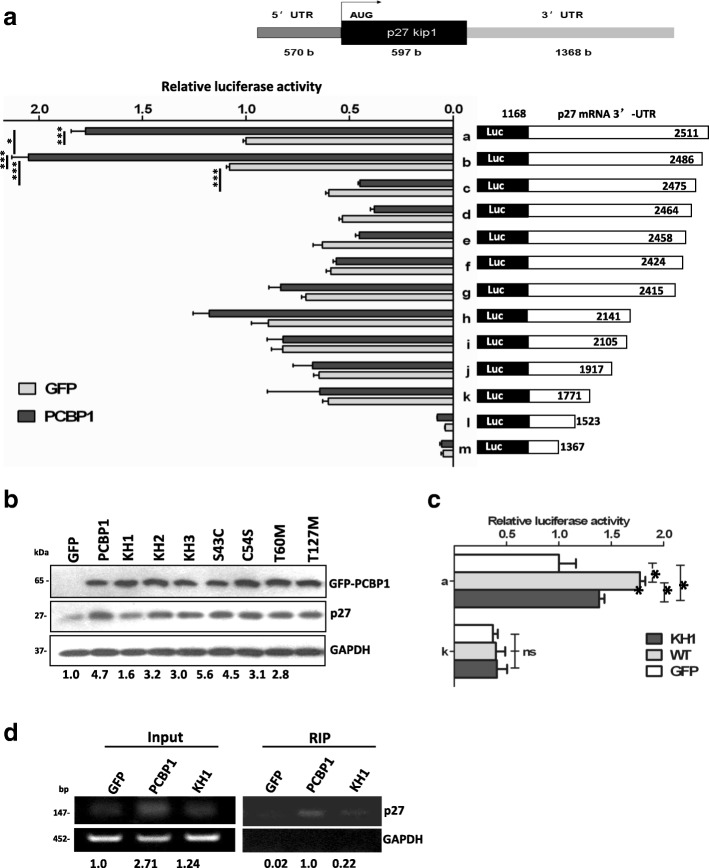
PCBP1 interacts with p27 mRNA 3'-UTR mainly through its KH1 domain. (**a**) Luciferase activities induced by the full-length p27 3'-UTR (construct a) and its serial deletions (construct b-m) in PCBP1 over-expressing cells or the control cells. Top panel shows the schematic diagram of human p27 mRNA. Full-length p27 mRNA 3'-UTR fragment and its deleted mutants were fused downstream of luciferase coding region in pGL3 plasmid. A2780 cells were transiently co-transfected with the indicated constructs with pRL-TK plasmid for 24-48 h and lyzed for enzyme activity measurement. Relative luciferase activity (Firefly/Renilla) was normalized from three independent experiments (mean±SD). (**b**) Immunoblot of p27 protein expression affected by the indicated PCBP1 mutations. A2780 cells transiently transfected with the pEGFP vector (Lane 1) or plasmids coding the wild-type PCBP1 (Lane 2) or its mutations (Lane 3-9) as indicated. The protein band intensity was scanned and measured by Image J software. Relative p27 level against GAPDH is further normalized against GFP and shown at the bottom. Results are representative of at least three independent experiments. (**c**) Effect of PCBP1 KH1 domain to Luciferase activity. A2780 cells were triply co-transfected with luciferase reporter containing full-length p27 3'-UTR (construct a) and plasmids encoding GFP-PCBP1 KH1 mutant, GFP-PCBP1, or GFP with pRL-TK, respectively. 48h after transfection, the enzyme activates were measured as in (**a**) Meanwhile, the reporter plasmid without PCBP1-binding p27 3'-UTR region (construct k), as a negative control, was similarly used for the above triple transfection. Relative luciferase activity (Firefly/Renilla) is presented from three independent experiments (mean±SD). * and ** represent *p*<0.05 and 0.01, respectively. ns indicates no difference. (**d**) RT-PCR detection of p27 mRNA bound with wild-type PCBP1 and the KH1 mutant. The experiments were conducted as in Fig. 1. The input or bound p27 mRNA was quantified based on band intensity and shown under each lane

As a RNA binding protein, PCBP1 has been recognized to enhance transcription, translation or repress translation by binding to mRNAs within the untranslated regions [5, 6], mostly in the 3’-UTRs. So we first focused on 3’-UTR of p27 mRNA. A series of deletions of p27 mRNA 3’-UTR were inserted into downstream of luciferase reporter to map whether p27 mRNA 3’-UTR binds to PCBP1 in PCBP1-overexpressing cells. Reporter analyses demonstrated that the lucifearase activity induced by the full length p27 3’-UTR exhibited approximately 1.8-fold higher in PCBP1-overexpressing cells, compared to that in GFP control counterparts. Likewise, deletion of nucleotides (nt) 2487 to 2511 in p27 3’-UTR did not affect the luciferase activity extent. In contrast, deletions of nt 1368 to 2475 in p27 3’-UTR evidently reduced the luciferase activity to the level in GFP control cells (Fig. 2a). These data suggested that PCBP1 plays an important role in upregulation of p27 protein by binding to the nt 2476–2485 within p27 3’-UTR, which is 5’-AUUAAGUAAU-3’, an AU-rich motif.

We next sought to address which part of PCBP1 is involved in interacting with p27 mRNA. It is perceived that KH domains [12, 36, 37] and phosphorylation status [16, 36, 37] of PCBP1 are important in its binding capability to proteins and mRNAs. We thus generated 7 PCBP1 mutations with single amino acid substitution to break its KH1 (G30A), KH2 (G114A) and KH3 (G296A) domains, phosphorylating sites (S43C, C54S,T60 M, T127 M) [27], respectively, to figure out the binding portion of PCBP1 to p27 mRNA. Immunoblots indicated that breakdown of PCBP1 KH1 domain clearly caused around 50% of p27 expression reduction, compared to that with wild-type PCBP1 (Lane 3 vs Lane 2, Fig. 2b) in all assayed mutants. However, mutation of the phosphorylation site of PCBP1 at S43 increased p27 protein level (S43C, Lane 6, Fig. 2b), which is in line with the view that phosphorylated PCBP1 losses its mRNA binding ability [16]. In contrast, mutations of the other two phosphorylation sites at T60 and T127 caused slight decrease of p27 level (Lanes 8, 9, Fig. 2b). Overall, our results indicated that the PCBP1 KH1 domain and the phosphorylation status would be important in PCBP1 binding to p27 mRNA, and subsequently regulating p27 protein level.

To further corroborate the effect of KH1 domain in RNA binding, we transfected the luciferase reporter fused with full length p27 3’-UTR (construct a) or its AU-rich motif-binding site deletion (k) into cells with PCBP1- or its KH1 mutant-overexpressing cells, respectively. Results revealed that, with co-transfected luciferase with full-length p27 3’-UTR, PCBP1 KH1 mutant overexpression evidently compromised the luciferase activity at least to 50% of that induced by wild-type PCBP1 overexpression (Fig. 2c, a), suggesting that PCBP1 KH1 domain is very crucial for PCBP1 binding to p27 mRNA. When co-transfection with the deletion of PCBP1 binding sequence AU-rich motif in p27 3’-UTR, luciferase activities in either wild-type or the KH1 mutant PCBP1 overexpression cells were reduced to the basal level as controls (Fig. 2c, k), in turn verifying the essential role of AU-rich motif in p27 mRNA 3’-UTR in PCBP1 binding. To clearly demonstrate the importance of PCBP1 KH1 domain in the binding, we conducted the RIP in PCBP1-overexpressing and its KH1 mutant cells and observed that KH1 mutation clearly attenuated PCBP1 binding ability to half of its wild-type (Fig. 2d), which is in line with the above luciferase activity results. Taken together, our results revealed that PCBP1 interacts with p27 mRNA mainly through its KH1 domain via an AU-rich motif located in p27 mRNA 3’-UTR.

### PCBP1 arrest cell cycle and enhance apoptosis under drug-induced stress

**Fig. 3:**
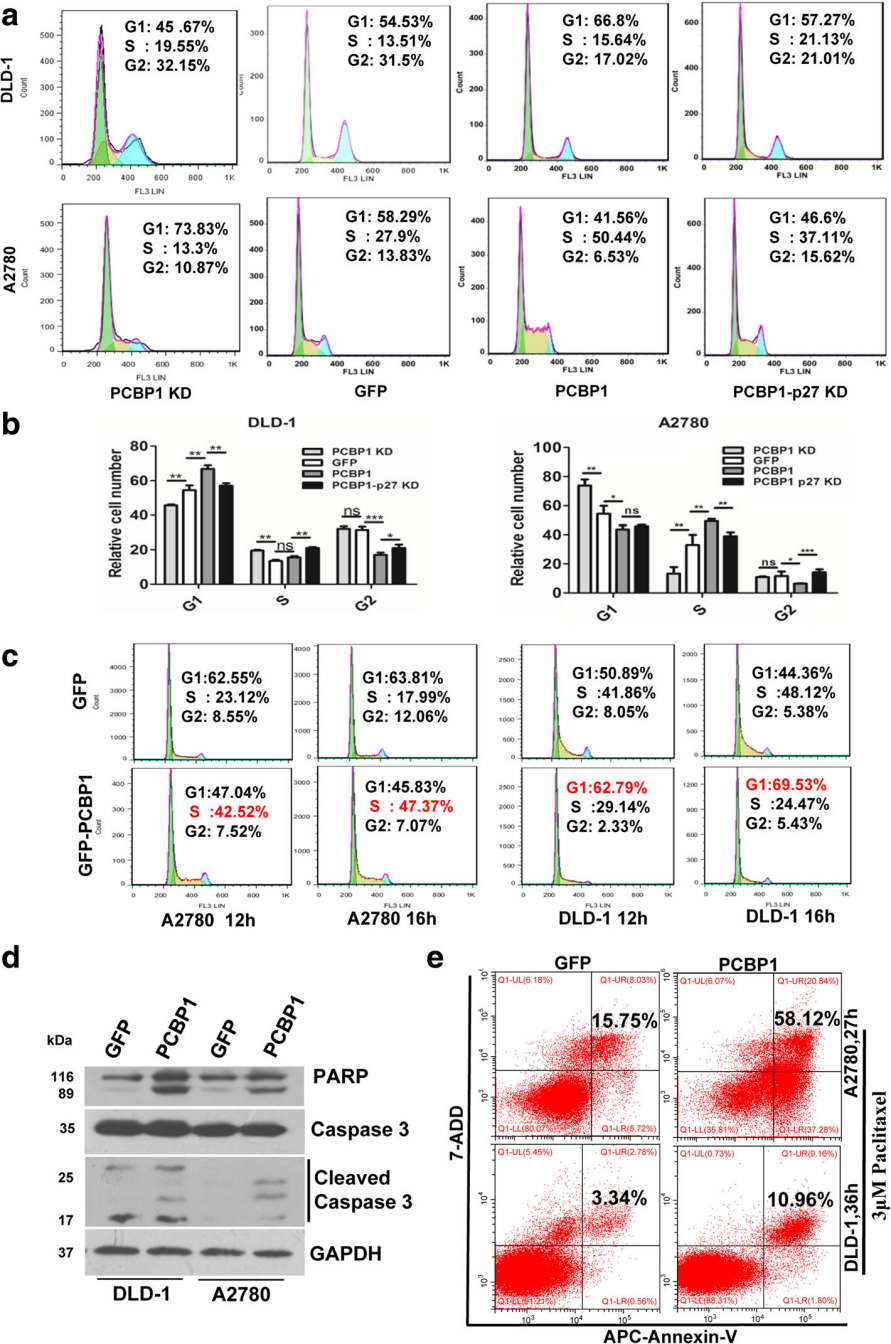
Overexpression of PCBP1 represses cell cycle progression and promotes apoptosis. (**a**) Flow cytometry analysis of cell cycle progression in A2780 and DLD-1 cells stably transfected with PCBP1 or PCBP1 with additional p27 knockdown. (**b**) The effect of PCBP1 and p27 in cell cycle progression of DLD-1 and A2780 cells. The populations of cells in G1, S and G2 phases are presented based on three independent experiments. *P <0.05, **P <0.01, ***P <0.001, ns indicates no difference (**c**) Flow cytometry analysis of cell cycle progression in A2780 and DLD-1 cells stably transfected with PCBP1 and the control cells treated with 0.08 μM MTX plus serum-free starvation for 24 h, and the whole medium was added for another 12 and 16 hours, respectively, for cell cycle progression analysis. (**d**) Representative immunoblot of the cleaved caspase-3 in PCBP1 overexpressing A2780 and DLD-1 cells. Cells were treated with 3 μM Paclitaxel for 24 hours in advance of immunoblotting analyses. (**e**) Flow cytometric analyses of apoptotic cells in A2780 and DLD-1 cells with PCBP1 overexpression, compared with their control cells. Cells were treated with 3μM Paclitaxel for the indicated time. Total percentage of Annexin V-positive cells is shown. Results are representative data from three independent experiments

p27 is known to induce cell cycle arrest, considering p27 expression is elevated by PCBP1, we investigated the function of PCBP1 in cell cycle progression. PCBP1 overexpression induced evident cell arrest in G1 phase of DLD-1 cells, and the additional p27 knockdown in the same cells counteracted this inhibitory effect, while PCBP1 knockdown alone also released the G1 arrest (Fig. 3a, b DLD-1). Unexpectedly, we found that PCBP1 overexpression in A2780 cells arrested cell population in S phase, and PCBP1 knockdown led to the opposite outcome, while additional p27 knockdown in PCBP1-overexpressing cells reversed PCBP1-induced effect, confirming the cell cycle inhibition of PCBP1 through p27 induction (Fig. 3a, b, A2780). To further clarify whether PCBP1 inhibit cell cycle at S phase in A2780 cells due to the incomplete cell synchronization, we treated the cells with serum-free starvation plus MTX treatment for 24 h, and then added the whole medium for another 12 and 16 h, respectively. Results demonstrated that PCBP1 arrested the cell cycle at S phase in A2780 cells (S phase: 42.52% at 12 h vs 47.37% at 16 h), compared to that of control cells (S phase: 23.12% at 12 h vs 17.99% at 16 h), while the G1 arrest in DLD-1 cells by PCBP1 was still observed (Fig. 3c). To double check the difference of cell phase inhibition between DLD-1 and A2780 cells by PCBP1, we did EdU staining to detect the cell proliferation status. Both immunofluorescence and flow cytometric results revealed that the proportion of EdU-positive cells with PCBP1-overexpression was more than that of the control A2780 cells, whereas clearly less in DLD-1 cell line (Additional file 4: Figure S2), which is in consistent with the results of cell cycle analyses, indicating the possible cell type-dependent inhibitory effect of PCBP1 in cell cycle progression. Indeed, p27 has functions in inhibiting both G1/S and G2/M cell cycle progression [38, 39], but more often in G1/S transition.

Considering that when cell cycle is inhibited, the cells would either go to apoptosis or become dormant under stress, we next sought to check whether PCBP1 exerts apoptotic effect under anticancer drug-induced stress via the cleaved Caspase-3 activation, as this activation has been acknowledged as a hallmark of apoptosis [40]. Caspase 3 is responsible for cleavage of nuclear enzyme poly (ADP-ribose) polymerase (PARP) and inactivates PARP during cell death. We treated the cells with paclitaxel and immunoblots manifested that the cleaved caspase-3 was overtly induced in both A2780 and DLD-1 cells by PCBP1 overexpression (Fig. 3d). As appearance of phosphatidylserine (PS) residues on a cell surface is an early event in apoptosis, Annexin V has a strong affinity to PS and is used as a probe of apoptosis. Flow cytometry analysis of PS and Annexin V showed that PCBP1 promoted cell apoptosis in both A2780 and DLD-1 cells at 3 μM paclitaxel (Fig. 3e). These results indicated that PCBP1 could sensitize cancer cells to anticancer drug-induced apoptosis.

### PCBP1 suppresses cell proliferation and tumorigenicity

**Fig. 4:**
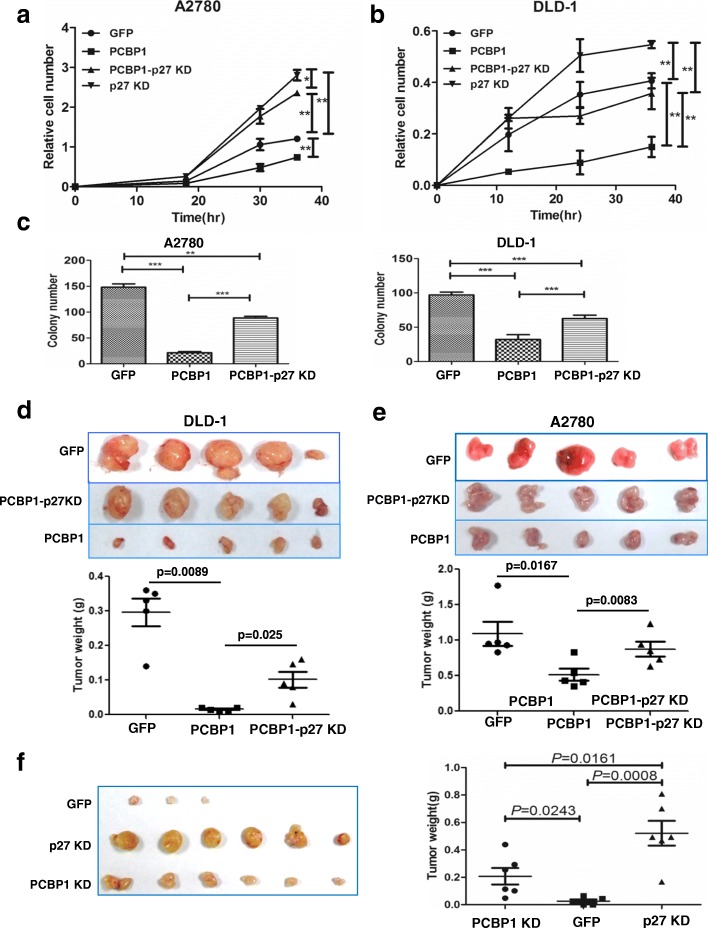
PCBP1 suppresses cell proliferation and tumorigenesis. (**a**, **b**) Cell viability analyses of A2780 (**a**) and DLD-1 (**b**) cells stably expressing PCBP1, PCBP1-p27 KD, p27 KD or GFP control by MTT assays. Data are presented as mean±SD (**P <0.01 by a 2-tailed Student’s t test). (**c**) Statistical analysis of colonies induced by A2780 or DLD-1 cells overexpressing PCBP1, PCBP1-p27 KD, or GFP control in soft agar assay for anchorage-independent growth. **P <0.01, n=3, two-tailed Student’s t test. (**d**, **e**) Tumor formation induced by DLD-1 (**d**) and A2780 cells (**e**) with PCBP1 overexpression plus p27 knockdown or PCBP1 alone, respectively. Cells were injected subcutaneously into flank sides of nude mice armpit. Tumors induced were harvested and weighed at 3 weeks post-injection by A2780 cells or at 4 weeks by DLD-1 cells. The xenograft tumor weights were analyzed and shown in the lower panel, respectively, *n*=5. (**f**) Tumor formation induced by DLD-1 cells with knockdown of either p27 or PCBP1. Cells were injected subcutaneously as above. Tumors were harvested at the 3rd week, weighted and analyzed. P values are indicated, *n*=5

The observation of cell cycle arrest by PCBP1 prompted us to determine its physiological impact in cell proliferation. Cell proliferation kinetics assays illustrated that PCBP1 overexpression significantly inhibited cell growth in both A2780 and DLD-1 cells, and this event was antagonized by the additional p27 knockdown (Fig. 4a, b and Additional file 5: Figure S3). In vitro soft-agar tumorigenicity assay also showed that PCBP1 overexpression obviously interrupted colony formation of both A2780 and DLD-1 cells, and the additional p27 silence almost blocked this event in the same cells (Fig. 4c, Additional file 6: Figure S4), indicating the upstream role of PCBP1 through p27 upregulation to suppress tumorigenesis.

To further verify the hierarchical relation between PCBP1 and p27 in tumorigenesis in vivo, we subcutaneously injected the PCBP1-overexpressing DLD-1 cells into nude mice and the result showed that PCBP1 inhibited tumorigenesis (Additional file 7: Figure S5), which is similar to our previous observation in HCT-116 cells [6]. Moreover, additional depletion of p27 in DLD-1 cells with PCBP1 overexpression disrupted the tumor-suppressive effect of PCBP1, leading to significantly accelerated tumor formation (Fig. 4d). Similarly, the secondary knockdown of p27 blocked the suppressive effect of PCBP1 in the A2780 cells with PCBP1 overexpression (Fig. 4e), indicating that PCBP1 suppresses tumorigenesis through p27 upregulation. To further assess if PCBP1 has non-p27 effect on tumor formation, we individually silenced PCBP1 and p27 expression in DLD-1 cells and conducted the tumor formation in vivo. Our results showed that although silence of PCBP1 clearly promoted tumor formation, while p27 knockdown resulted in more aggressive tumorigenesis than PCBP1 knockdown in DLD-1 cells (Fig. 4f), indicating the multiple regulation and the key role of p27 in tumorigenesis. Considering all above results together, we concluded that PCBP1 inhibits tumorigenesis through upregulation of p27 expression.

### PCBP1 up-regulates p27 by stabilizing and enhancing p27 mRNA translation

**Fig. 5:**
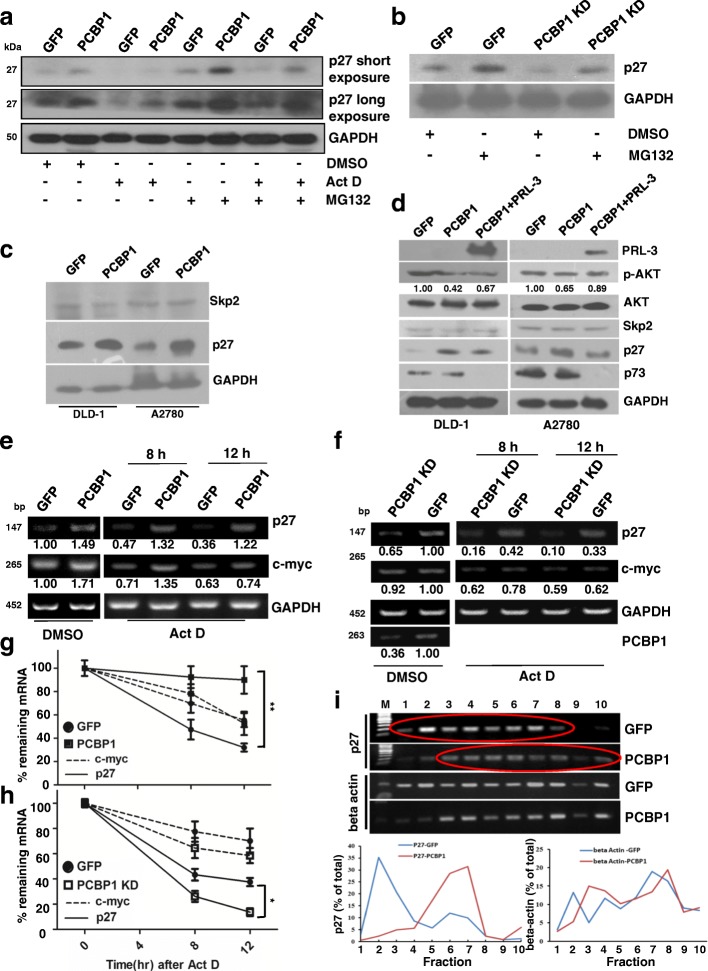
PCBP1 up-regulates p27 expression by enhancing the mRNA stability and translation. (**a**) Immunoblot of endogenous p27 protein levels upon transcription inhibition or/and proteasome degradation repression. A2780 cells overexpressing GFP-PCBP1 or GFP alone were treated with DMSO, Act D at 0.5 μg/ml for 8 h, MG132 at 20 μM for 4 h, or with their combination for 4 h, and analyzed. Results are representative of at least three independent experiments. (**b**) Immunoblot of endogenous p27 protein levels in A2780 cells with endogenous PCBP1 knockdown. Cells were treated with MG132 as in (**a**) (**c**) Immunoblot of p27 ubiquitin ligase Skp2 and p27 expression in the indicated cells. No evident Skp2 expression alteration is shown. (**d**) Immunoblot of the indicated proteins possibly related to p27 protein expression and cell survival. (**e**) Semi-quantitative RT-PCR analysis of p27 or c-myc mRNA stability in A2780 cells overexpressing PCBP1 or GFP control, on condition of Act D treatment to terminate the novel mRNA transcription. c-myc and GAPDH were used as positive and negative controls. The PCR of p27 were performed with 20 cycles, c-Myc with 25 and GAPDH for 20 cycles, respectively. Relative p27 and c-myc level against GAPDH were further normalized against DMSO treated GFP and shown at the bottom. (**f**) Semi-quantitative RT-PCR analyses of p27 or c-myc mRNA stability in A2780 cells with endogenous PCBP1 knockdown by specific shRNAs as the method in E. *p<0.05 (**g**, **h**) Analyses of p27 or c-myc mRNA duration in the cells as shown in (**e**, **f**) Band intensities were measured by ImageJ software and normalized against their corresponding GFP-Lane treated with DMSO. All experiments were independently repeated at least for 3 times. **p<0.01 (I) Ribosome profiling of p27 mRNA. Major percentage of p27 mRNA cells is located in the lighter polysome fractions (1–8) of the parental DLD-1, while overexpression of PCPB1 results in a shift of p27 mRNA to the heaver fractions (3–10). Beta-actin mRNA is shown as an internal negative control. The marked numbers are equivalent to the collected fractions after gradient centrifuge. Fraction 1 represents the top (10%) of the gradient, fraction 10, the bottom (50%) of the gradient

Given the tumor suppression effect of PCBP1 through p27 upregulation, we sought to unravel the underlying mechanism of p27 upregulation by PCBP1. We first checked whether the p27 increase was resulted from the enhanced mRNA transcription by stopping the new mRNA synthesis with Actinomycin D (Act D). Results showed that although the overall p27 protein amounts in both control and PCBP1-overexpressing cells were clearly decreased due to the general reduction of new mRNAs, but the relative p27 protein level still elevated in PCBP1-overexpressing cells, indicating the posttranscriptional regulation of p27 (Lanes 3–4, Fig. 5a). As p27 can be specifically degraded by E3 ligase Skp2-mediated proteasome degradation, we next treated the cells with a proteasome inhibitor MG132, and the immunoblots demonstrated that p27 protein was still more in the PCBP1-overexpressing cells, hinting the authentic upregulation of p27 by PCBP1, beyond the ubiquitin-mediated degradation (Lanes 5–6, Fig. 5a). Furthermore, the combined treatments with Act D and MG132 resulted in the similar relative ratio of p27 protein amount in parental to PCBP1-overexpressing cells (Lanes 7–8, Fig. 5a). In contrast, knockdown of endogenous PCBP1 led to the reduced p27 expression, irrespective of p27 degradation inhibition (Fig. 5b). Taken together, our results indicated that PCBP1 upregulates p27 at post-transcriptional level, although both general transcription and protein degradation effecting p27 expression exist.

We previously showed that PCBP1 delays the translation of the metastatic phosphatase PRL-3 that can activate the AKT and maybe the subsequent Skp2 to modulate p27 expression. To clarify this possibility, we investigated the effect of PCBP1 to Skp2 expression and showed that, even in the DLD-1 cell with non-detectable PRL-3 expression as we formerly shown [6], PCBP1 did not influence Skp2 expression, as well as that in PRL-3 positive A2780 cells (Fig. 5c), suggesting a Skp2-independent manner of PCBP1 to p27 upregulation. To further confirm it, we supplementally expressed PRL-3 in PCBP1-overexpressing cells and observed a slight Skp2 increase, but the p27 protein level still higher than that in control cells, while the PRL-3 effect in AKT activation and cell survival by inhibiting p73 expression still exists to some extend (Fig. 5d). Thus, our results showed that PCBP1 would mainly upregulate p27 expression via the association to p27 mRNA.

RNA-binding proteins can increase mRNA amount by modulation of mRNA stability. Likewise, we wonder whether PCBP1 can stabilize p27 mRNA to result in the higher protein expression, as PCBP1 stabilizing many other mRNAs, such as α-globin [41], tyrosine hydroxylase [42] and erythropoeitin [43]. Our result demonstrated that p27 mRNA in PCBP1-overexpressing cells was more stable than that in control cells up to 12 h, when the newly synthesized transcripts were blocked by Act D (Fig. 5e). Time course tracing of RNA turnover revealed that PCBP1 overexpression dramatically increased the half-life of p27 mRNA, compared to the previously known PCBP1-bound c-Myc [11] (Fig. 5g and Additional file 6: Figure S5A). Conversely, knockdown of endogenous PCBP1 accelerated p27 mRNA degradation by shortening p27 mRNA half-life from 8.35 h to 6.21 h (Fig. 5f, h and Additional file 6: Figure S5B).

PCBP1 also acts as translational modulator [27]. To understand whether PCBP1 additionally affects p27 mRNA translation, we analyzed the ribosome profile of p27 mRNA in parental and PCBP1 overexpressing DLD-1 cells. Results showed that in parental cells, p27 mRNA was found mainly in the lighter polysome fractions (Fig. 5i, upper: 2–8, red circle). When PCBP1 was overexpressed, p27 mRNA was shifted into heavier polysome fractions (Fig. 5i, lower: 3–10, red circle), indicating it was efficiently translated into protein.

Considering PCBP2 is highly homologous to PCBP1, to clarify whether PCBP2 can similarly affect p27 protein expression, we knocked down PCBP2 expression with two specific siRNAs in both parental and PCBP1-overexpressing cells (Additional file 8: Figure S6A), and did not observe significant effect of PCBP2 to p27 in mRNA and protein levels (Additional file 8: Figure S6B). Collectively, these results suggest that PCBP1 can specifically up-regulate p27 protein level by stabilizing p27 mRNA stability and enhancing its translation.

### Loss of PCBP1 accompanies p27 downregulation in carcinomas

**Fig. 6:**
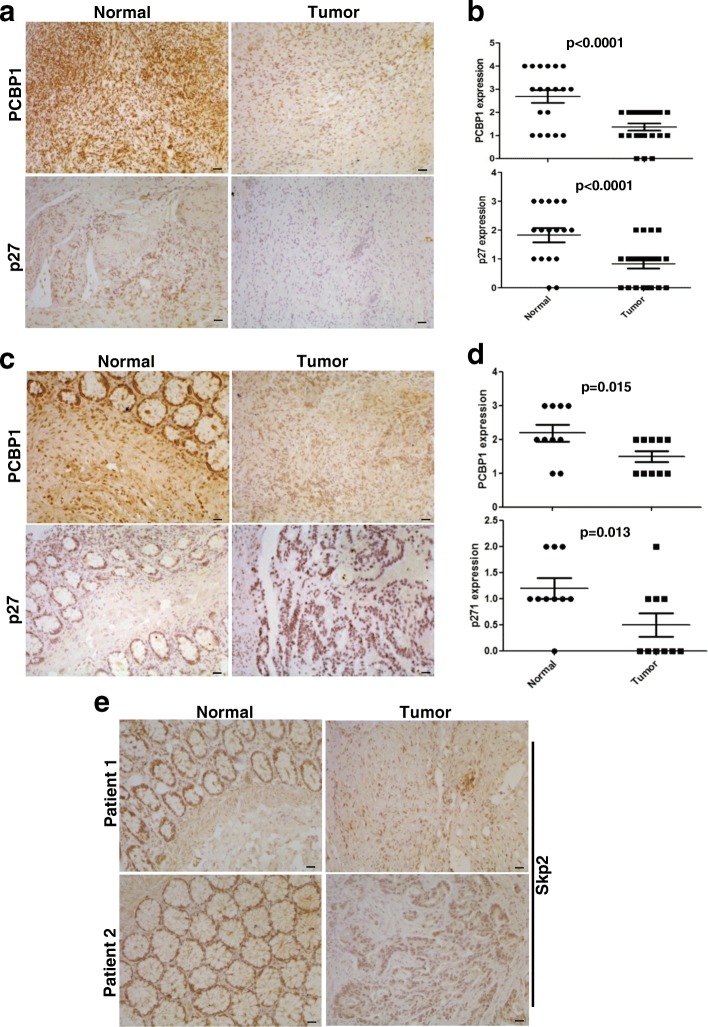
Relevance of PCBP1 to p27 expression in human cancer samples. (**a**) Representative IHC staining of PCBP1 and p27 protein expression in tumor adjacent normal and tumor regions of ovary carcinoma. Scale bars are equal to 50 μm. (**b**) Statistical analysis of PCBP1 and p27 in normal and malignant tissues of ovary. 18 normal tissues and 21 carcinoma tissues were analyzed. The unpaired student t test was used to check the group difference. p values are shown. (**c**) Representative IHC staining of PCBP1 and p27 protein expression in the paired adjacent normal and tumor regions of colon carcinoma. Scale bars are equal to 50 μm. (**d**) Statistical analysis of PCBP1 and p27 expression in 10 paired fresh normal and malignant tissues of colon tissues. The paired student t test was carried out to check the group difference. p values are shown. (**e**) Representative IHC staining of p27 ubiquitin ligase, Skp2 protein expression in tumor adjacent normal and tumor regions of colon carcinoma. No evident Skp2 expression difference was observed. Scale bars are equal to 50 μm

To validate whether PCBP1 and p27 expression are correlated with malignancies and have predictive values to diagnosis, we clarified the relationship between PCBP1 and p27 expression in both ovary and colon tumor tissues. Immunohistochemistry showed that lower expression of PCBP1 and p27 were synchronously observed in both ovary (Fig. 6a, b; Additional file 9: Figure S7) and colon (Fig. 6c, d; Additional file 10: Figure S8) carcinomas. Given that PCBP1 mainly regulates p27 expression at mRNA level, we compared the paired normal and tumor tissues of colon samples and found that p27 mRNA level was indeed decreased in the PCBP1 low expression tumors (Additional file 11: Figure S9A), validating our results from tumor cells.

To confirm our result in a broad spectrum, we searched dataset of human renal caner and examined PCBP1 and p27 expression. Results showed the similar physiological significance of PCBP1 in renal tumors to ovary and colon carcinoma analyzed above (Additional file 12: Figure S10A, C, D). A positive correlation between PCBP1 and p27 protein levels was also observed (Additional file 12: Figure S10B).

We also searched and found a GEO database related to ovary cancer. Analysis result indicated an obvious decreased p27 mRNA level in the paired ovary cancer epithelia cells, compared to that in the corresponding normal tissue cells, without p27 ligase Skp2 mRNA changing (Additional file 11: Figure S9B, GDS3592). Similarly, we observed there was no evident Skp2 protein level change in our analyzed colon tumor samples either (Fig. 6e), hinting there are a subset of tumors with low p27 and Skp2 expressions, which also indicating the p27 downregulation is not resulted from high Skp2-mediated degradation. The Cancer Genome Atlas (TCGA) data also showed the very rare amplification of Skp2 in both ovary and colon cancers (Additional file 11: Figure S9C).

Furthermore, we also searched The Human Protein Atlas database and found a set of renal tumors, in which patients with low PCBP1 level more likely show the higher tumor stage and poor five-year survival rate (Additional file 1: Table S5 and Additional file 12: Figure S10E), indicating that PCBP1 could be a novel predictor of prognosis in cancer patients.

Moreover, GEO data analyses also indicated that simultaneous downregulation of PCBP1 and p27 mRNA is positively related to anticancer drug resistance, including to Tamoxifen, Doxorubincin, and Lapatinib in breast cancer cells (GSE26459, GSE24460, GSE16179) [33–35], respectively (Additional file 13: Figure S11), suggesting the prognostic role of PCBP1 through p27 in cancer therapy.

## Discussion

Loss of PCBP1 is involved in various carcinogenesis [6, 13, 25, 26]. Recently, PCBP1 has been shown as a negative regulator of thyroid carcinoma [44], a negative regulator of EMT related to metastasis in NSCLC [45], while PCBP1 expression is suppressed in peritoneal gastric cancer metastasis [46]. PCBP1 also can sensitize colorectal cancer to anti-cancer drug [47]. All these highlight the clinical significance of PCBP1 in cancers. In our previous work, we also disclosed that PCBP1 inhibits AKT activation through suppression of metastasis-associated PRL-3 [6, 11, 12], but the correlation between PCBP1 and PRL-3 expression is not so tight, suggesting its PRL-3-independent ways. Thus, investigation of the alternative PCBP1 function in tumorigenesis would unravel other unknown events which would be of help to tumor diagnosis and therapy. Here we captured the PCBP1-bound mRNAs in transcriptome-wide with RIP-seq strategy. Our results exhibited that multiple mRNAs can be enriched in human A2780 cells (Fig. 1a and Additional file 1: Table S4), some of which have been identified as PCBP1 binding targets, including p21 and eIF4E [36, 48] that are involved in tumorigenesis. Meanwhile, our results indicate the multiple binding capabilities of PCBP1 and hint the aberrant PCBP1 expression would affect a large spectrum of gene expression, resulting in many physiological abnormities in a specific context, including transformation.

Among the enriched transcripts, p27 mRNA is a novel PCBP1-bound transcript related to cell cycle proliferation, whose abnormal expression is putatively described in many types of cancers. Our results clearly demonstrated that PCBP1 at least binds to the 3’-UTR of p27 mRNA to evidently block the p27 mRNA degradation, leading to higher p27 protein level, although some other indirect possibilities are existed. PCBP1 can translationally repress PRL-3 expression to activate AKT [6, 11, 12], which further to regulate p27 and its ubiquitin ligase Skp2. However, in a subset of breast carcinomas, it has shown that loss of p27 expression is an independent predictor of both overall survival and disease-free survival, as Skp2 is expressed at low levels despite low expression of p27 [49]. Our results here may unveil the puzzle that loss of PCBP1 destabilizes p27 mRNA at the first step to lead to low p27 expression, irrespective of Skp2 levels and the posttranslational regulation via other manners (Fig. 5c, d), since Skp2 expression is rarely seen to be upregulated in the detected tumor samples (Fig. 6; Additional file 11: Figure S9C). As a RNA-binding protein, PCBP1 can directly regulate gene expression in posttranscription by binding to 3’-UTR (e.g. *AR, p21, p63, eNOS, POLH*) [7–10, 50] or 5’-UTRs (e.g. *c-myc, PRL-3, EV71*) [6, 11, 12] of various mRNAs, although the molecular details of such regulation remain to be structurally determined.

It has been reported that RNA binding proteins, including HuR [51–53], DND1 [54, 55], RBM38 [56], CRD-BP [57, 58] and hnRNP E2 [59], can compete same binding sites located on their correspondingly targeted genes with microRNAs, subsequently antagonizing the microRNA-targeted gene silencing. DND1 binds 3’-UTR of p27 mRNA to block the binding sequences targeted by p27 specific microRNAs, and maintains p27 protein expression in a germ-cell tumor cell line [54]. Thus, PCBP1 may work in the same manner to compete to the binding of some microRNAs with p27 mRNA and cause the less mRNA degradation.

Our results revealed that break of PCBP1 KH1 domain apparently mitigated p27 protein expression and the reporter activity, but not the KH2 and KH3 mutations, indicating that PCBP1 binds to p27 mRNA mainly through its KH1 domain, rather than KH2 and KH3 domains, which is consistent to that PCBP1 KH1 domain interacts with stem-loops I and IV of EV71 5’UTR, facilitating viral replication [12]. Our results also pointed that mutation of PCBP1 phosphorylation sites at T60 and T127 led to lower p27 protein level, which is opposite to the mutation of S43 that can block PCBP1 mRNA-binding ability [16]. Therefore, our results could also indicate the complicated regulatory roles of PCBP1 phosphorylation in its RNA-binding capability.

p27 is a putative inhibitor of cyclin E-CDK2 [60–62] and binds cyclin E/A:CDK2 complex to inhibit G1-S transition [63]. Usually, p27 accumulates on cell cycle exit, and is rapidly degraded when cells re-enter the cell cycle from quiescence [64]. Our results showed that cell cycle was arrested in G1 phase of DLD1 cells, while in S-G2 phase of A2780 cells (Fig. 3a-c). These arrested states can be released by the additional knockdown of p27 in the both cell lines, manifesting that p27 mRNA stabilization by PCBP1 may instinctively maintain p27 protein at high level for longer time to thoroughly check the cell cycle stages and integrity. Our results also illuminate that cell cycle modulation by p27 associated to PCBP1 is independent of the previously recognized G1/S transition checkpoint, as in 2780 cells, PCBP1 clearly block cell cycle at S phase (Fig. 3a-c; Additional file 3: Figure S2). Indeed, it is already known that p27 has function in inhibiting both G1/S and G2/M cell cycle progression [38, 39]. Therefore, it would be particularly interested in exploring what is the exact downstream event of PCBP1-p27 signaling in maintaining appropriate cell cycle progression.

It is believed that PCBP1 can bind to multiple transcripts, such as POLH (DNA polymerase η) to control its expression via mRNA stability [50], and POLH loss is responsible for the human cancer-prone syndrome XPV, but there is no in vivo and clinical result to further support this notion. Here, our both in vitro and in vivo results implied that p27 could be likely the most effective downstream target of PCBP1 in inhibition of tumorigenesis, since the cell proliferation and tumorigenesis inhibition imposed by PCBP1 can almost be blocked by the additional knockdown of p27 in the same cells. Clinical patient samples also validated this PCBP1-p27 positive correlation, that is, loss of PCBP1 simultaneously accompanies the downregulation of p27 in tumors, which further confirms that the signaling of PCBP1 through p27 plays critical role in tumorigenesis inhibition. As a binding target of PCBP1 and cell cycle family member, p21 could be also involved in PCBP1-dependent cell cycle inhibition. But based on our results, additionally silencing p27 in PCBP1-overexpressing cells can almost neutralized PCBP1’s effect, indicates the dominant PCBP1-p27 signaling in tumor suppression.

Given that tumors with p27 downregulation have poor prognosis [65, 66], including colon, ovary and breast cancers, and p27 is rarely inactivated in human cancers, our results can suggest that loss of PCBP1 most likely could be the key initial step for p27 downregulation, leading to eventual tumorigenesis, as we tentatively used various types of tumor cells as well as tumor datasets in this study to draw the similar conclusion, hinting the general role of PCBP1-p27 signaling in tumorigenesis suppression.

## Conclusion

**Fig. 7:**
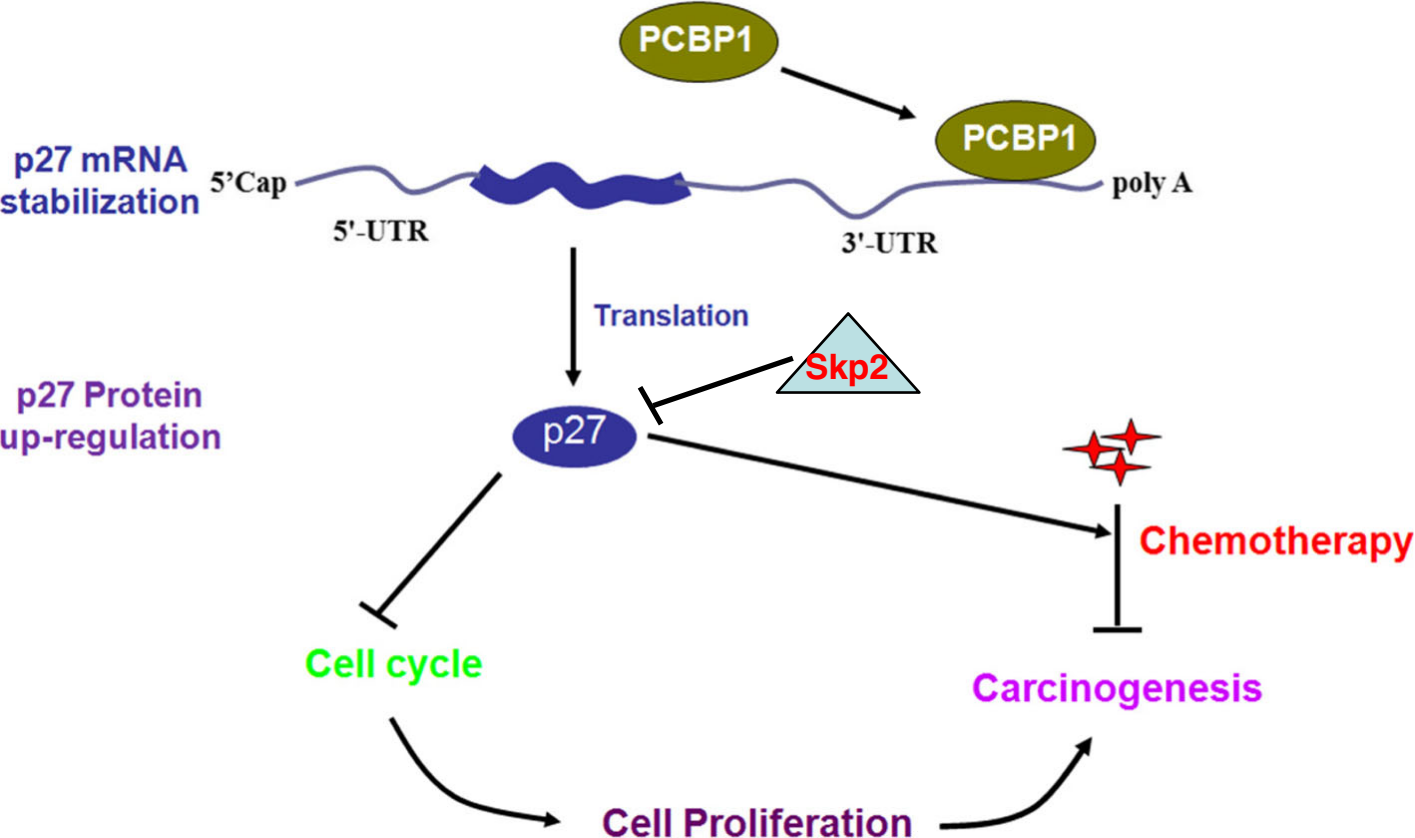
Proposed model of PCBP1 stabilizing p27 mRNA to repress cell transformation and carcinogenesis. PCBP1 binds to p27 mRNA 3'-UTR region via 5'-AUUAAGUAAU-3' to stabilize mRNA against degradation, while enhance the translation, resulting in more p27 protein that participates in cell cycle surveillance to maintain cell homeostasis and to inhibit carcinogenesis, while p27 degradation by ubiquitin ligase Skp2 promotes cell cycle progression after the proper surveillance. In tumor tissues, anti-cancer drugs can induce p27 expression to induce tumor cell cycle arrest and apoptosis. Loss of PCBP1 expression would expose p27 mRNA for degradation, leading to lower expression of p27 protein and unregulated cell proliferation, eventually hyperplasia and carcinogenesis, which are independent of Skp2 status

In summary, our study indicates that PCBP1 binds to p27 mRNA to stabilize and simultaneously enhance p27 mRNA translation like PABPs as a CAP-binding protein or other unknown manners to coordinately upregulate p27 protein level, consequently inhibiting cell cycle progression, promoting apoptosis and repressing tumorigenesis, especially in the transformed tissues with Skp2 silence (Fig. 7). Loss of PCBP1 in tumors induced p27 mRNA degradation to release cell cycle progression, leading to transformation and carcinogenesis. Therefore, PCBP1 could be a novel marker for cancer diagnosis and therapy, and the tumor cell cycle inhibition strategy would be of help to the therapy of PCBP1-depleted tumors.

## Additional files

Additional file 1: **Table S1.** Primers for RT-PCR. **Table S2.** Primers for p27 3’UTR series deletion. **Table S3.** Primers for PCBP1 mutations. **Table S4.** Part of mRNAs identified in GFP-PCBP1-RIP only. **Table S5.** Correlation between clinicopathologic features with PCBP1 expression in renal cancer patients. (DOCX 23 kb)

Additional file 2: Supplementary Experimental Procedures. (DOCX 19 kb)

Additional file 3: **Figure S1.** PCBP1 increased p27 mRNA stability in A2780, DLD-1 and MDA-MB-231 cells. (A). Schematic procedure of isolation and identification of PCBP1-bound RNA transcripts. (B). Semi-quantitative RT-PCR detection of p27 mRNA levels in the indicated cell lines. A positive correlation between PCBP1 and p27 mRNA level is observed in these cell lines. (PPT 740 kb)

Additional file 4: **Figure S2.** PCBP1 overexpression inhibits cell cycle progression. (A). Representative immunofluorescence staining of A2780 and DLD-1 cells with EdU and Hoechst in cells with endogenous PCBP1 knockdown (PCBP1 KD), GFP-PCBP1 overexpression (PCBP1) and the additional p27KD in PCBP1-overexpressing cells (PCBP1-p27KD). (B). Statistical analyses of EdU positive cells in A. The EdU positive cells were counted randomly in 3 views and analyzed. (mean ± SEM, *n* = 3 per group). (C). Representative flow cytometry analysis of A2780 and DLD-1 cells with PCBP1 overexpression after EDU staining. Ratios of EdU + GFP-positive cells to GFP positive cells or GFP-PCBP1-positive cells are shown in the right-upper quadrant of each diagram. (PPT 2149 kb)

Additional file 5: **Figure S3.** Knockdown efficiency of p27 in A2780 and DLD-1 cells. (A). Immunoblots of p27 in DLD-1 and A2780 cells upon its two specific shRNAs transfection after 46 h. (B) Immunoblots of p27 in the stably PCBP1-overexpressing DLD-1 and A2780 cells with supplemental p27 KD with two specific shRNAs. (PPT 2285 kb)

Additional file 6: **Figure S4.** Colonies induced by A2780 or DLD-1 cells overexpressing PCBP1. PCBP1, PCBP1-p27 KD and GFP control cells were analyzed by soft agar assay for anchorage-independent growth. Colonies were stained with Trypan Blue and photographed. (PPT 609 kb)

Additional file 7: **Figure S5.** p27 mRNA stabilized by PCBP1. The half-life of p27 mRNA was derived from the decay curve plotted from the Fig. 6g and h. Linear regression equation in A: PCBP1, y = − 0.0096× + 0.998,R2 = 0.99; GFP, y = − 0.058× + 0.9844, R2 = 0.9865; B: PCBP1 KD, y = − 0.0749× + 0.965, R2 = 0.9607; GFP, y = − 0.0749× + 0.9677, R2 = 0.9387. (PPT 309 kb)

Additional file 8: **Figure S6.** No effect of PCBP2 on p27 expression on both mRNA and protein levels. (A). RT-PCR analysis of PCBP2 knockdown efficiency by 2 specific siRNAs in A2780 GFP control cells or GFP-PCBP1 overexpressing cells. (B). Immunoblot analysis of p27 expression upon PCBP2 knockdown in A2780 GFP control cells or GFP-PCBP1 overexpressing cells. GAPDH was used as a loading control. (PPT 2665 kb)

Additional file 9: **Figure S7.** Expression of PCBP1 and p27 in ovary cancer samples compared to that in the normal tissues. The indicated protein expression level was defined based on the staining intensity under the same robust IHC staining condition. (PPT 1277 kb)

Additional file 10: **Figure S8.** Expression of PCBP1 and p27 in paired colon cancer samples compared to that in the normal tissues. The indicated protein expression level was defined based on the staining intensity under the same robust IHC staining condition. (PPT 1072 kb)

Additional file 11: **Figure S9.** Relationship of PCBP1 to p27 mRNA level in tumor samples. (A). Semiquantitative RT-PCR detection of PCBP1, p27 mRNA expression in conlon tumor tissues. GAPDH was used as control. p27 mRNA level is correlated to PCBP1 level in the paired normal (N) and tumor (T) samples of colon tissues. (B). p27 mRNA level as well as its ubiquitin ligase Skp2 in ovarian epithelia. GEO ID is shown. (C). TCGA gene profile of Skp2 in tumors indicates the relative low amplification in ovary and colon tumors. (PPT 2633 kb)

Additional file 12: **Figure S10.** A positive correlation between PCBP1 and p27 proteinlevels in human renal cancer samples. (B) Relationship between PCBP1 and p27 protein expression in IHC staining. A positive correlation between PCBP1 and p27 protein levels is observed in tumors from 7 patients. (C) RT-PCR detection of PCBP1, p27 mRNA exprission in renal tumor tissues. GAPDH was used as control. (D) Immunoblot of PCBP1 and p27 proteins in healthy tissues (N), tumor adjacent region (A) and tumor tissues (T). GAPDH was used as control. E. Kaplan-Meier survival curve of renal carcinoma patients with different expression level of PCBP1. Data is from https://www.proteinatlas.org/ENSG00000169564-PCBP1/pathology/tissue/renal+cancer. (PPT 2557 kb)

Additional file 13: **Figure S11.** PCBP1 and p27 expression in tumor cells are correlated to anti-cancer drug sensitivity. PCBP1 and p27 expression were analyzed by data from GEO database, and high expression of PCBP1 and p27 were observed in Tamoxifen (GSE26459), Doxorubicin (GSE24460) and Lapatinib (GSE16179) sensitive breast cancer subclones (n indicates the analyzed cell subclone numbers). (PPT 92 kb)

## Abbreviations

3’UTR: 3’ untranslated region
Act D: Actinomycin D
FBS: Fetal bovine serum
GEO: Gene Expression Omnibus
hnRNP: Heterogeneous nuclear ribonucleoprotein
KH domain: K Homology domain
MTX: Methotrexate
nt: Nucleotide
p27: p27^Kip1^
PBS: Phosphate-buffered saline
PCBP1: Poly C Binding Protein 1
PRL-3: Phosphatase of Regenerating Liver-3
RIP: RNA immunoprecipitation
RT-PCR: Reverse Transcription-Polymerase Chain Reaction
shRNA: Short hairpin RNA
TCGA: The Cancer Genome Atlas
