# Supplementary material for: PCBP1 depletion promotes tumorigenesis through attenuation of p27Kip1 mRNA stability and translation
Source: J Exp Clin Cancer Res. 2018 Aug 7;37:187. doi: 10.1186/s13046-018-0840-1 (PMC6081911; doi:10.1186/s13046-018-0840-1)
Supplement: Supplementary file 1 — Table S1. Primers for RT-PCR. Table S2. Primers for p27 3’UTR series deletion. Table S3. Primers for PCBP1 mutations. Table S4. Part of mRNAs identified in GFP-PCBP1-RIP only. Table S5. Correlation between clinicopathologic features with PCBP1 expression in renal cancer patients. (DOCX 23 kb) [file 13046_2018_840_MOESM1_ESM.docx]

**Four supplementary tables:**

| **Table S1 Primers for RT-PCR** | | |
| --- | --- | --- |
| **Target gene for**  **Amplification** | **Direction of**  **Primer** | **Sequence (5′→ 3′)** |
| PCBP1 | Forward | CATCCGCTAAGAATTTAAAAAT |
|  | Reverse | AAGACAGCAATTCCCAGC |
| p27 | Forward | AGACGGGGTTAGCGGAGCAA |
|  | Reverse | TCTTGGGCGTCTGCTCCACA |
| p21 | Forward | GACTCTCAGGGTCGAAAACG |
|  | Reverse | CTTCCTGTGGGCGGATTAG |
| MYC | Forward | TGGTCTTCCCCTACCCTCTCAAC |
|  | Reverse | GATCCAGACTCTGACCTTTTGCC |
| GAPDH | Forward | ACCACAGTCCATGCCATCAC |
|  | Reverse | TCCACCACCCTGTTGCTGTA |

| **Table S2 Primers for p27 3’UTR series deletion** | | |
| --- | --- | --- |
| **Name** | **Direction of**  **Primer** | **Sequence (5′→ 3′)** |
| FL (1168-) | Forward | GC**TCTAGA**ACAGCTCGAATTAAGAATATGT |
| a (-2511) | Reverse | **GGATCC**AATAGCTATGGAAGTTTT |
| b (-2486) | Reverse | **GGATCC**GATTACTTAATGTGTAACAAT |
| c (-2475) | Reverse | **GGATCC**GTGTAACAATAATTGGCATCT |
| d (-2464) | Reverse | **GGATCC**ATTGGCATCTTTTTCACACAT |
| e (-2458) | Reverse | **GGATCC**ATCTTTTTCACACATTAC |
| f (-2424) | Reverse | **GGATCC**TCAGTATGCAACCTTTTAAGCAT |
| g (-2415) | Reverse | **GGATCC**CAACCTTTTAAGCATAGCCA |
| h (-2141) | Reverse | **GGATCC**TCTACATGAACCTTCCTTGAAAA |
| i (-2105) | Reverse | **GGATCC**GTGGATAAATGGACAGAGTA |
| j (-1917) | Reverse | **GGATCC**CATTCAAAACTCCCAAGCAC |
| k (-1771) | Reverse | **GGATCC**GCCACATGCAGCTATCTAACA |
| l (-1523) | Reverse | **GGATCC**CAAAGCAAGCTCTTCATACCC |
| m (-1367) | Reverse | **GGATCC**CCAACGCTTTTAGAGGCAGAT |

| **Table S3 Primers for PCBP1 mutations** | | |
| --- | --- | --- |
| **Name** | **Direction** | **Sequence (5′→ 3′)** |
| KH1/ ***G30A*** | Forward | AGCATCATTG**C**GAAGAAAGGGGAGTCGGTTAAGAG |
|  | Reverse | CCCTTTCTTC**G**CAATGATGCTTCCTACTTCCTTTC |
| KH2/  ***G114A*** | Forward | CCCTGATTG**C**GAAAGGCGGGTGTAAGATCAAAG |
|  | Reverse | CCGCCTTTC**G**CAATCAGGGAGCCGCACTGGGTG |
| KH3/  ***G296A*** | Forward | CTGCATAATCG**C**GCGCCAAGGCGCC |
|  | Reverse | CGCCTTGGCGC**G**CGATTATGCAGCC |
| S43C | Forward | CGAGGAG**T**GTGGCGCGCGGATCAACATCTC |
|  | Reverse | GCGCCAC**A**CTCCTCGCGGATCCTCTTAACC |
| C54S | Forward | GAGGGGAATT**C**TCCGGAGAGAATCATCACTC |
|  | Reverse | TCTCTCCGGA**G**AATTCCCCTCCGAGATGTTG |
| T60M | Forward | AGAATCATCA**TG**CTGACCGGCCCCACCAATGCCATC |
|  | Reverse | GGCCGGTCAG**CA**TGATGATTCTCTCCGGACAATTCC |
| T127M | Forward | GAGAGTA**T**GGGGGCGCAGGTCCAGGTGG |
|  | Reverse | CGCCCCC**A**TACTCTCGCGGATCTCTTTG |

| **Table S4 Part of mRNAs identified in GFP-PCBP1-RIP only** | | | |
| --- | --- | --- | --- |
| **Gene symbol** | **Gene description** | **GenBank accession** | **Rank/**  **Enrichment** |
| AP3D1 | Homo sapiens adaptor-related protein complex 3, delta 1 subunit | NM_001261826.1 | 11 |
| ANKRD13B | Homo sapiens ankyrin repeat domain 13B | NM_152345.4 | 9 |
| NOMO2 | Homo sapiens NODAL modulator 2 (NOMO2), transcript variant 2 | NM_001004060.1 | 9 |
| KIRREL | Homo sapiens kin of IRRE like (Drosophila), mRNA | NM_001286349.1 | 8 |
| ADCγ9 | Homo sapiens adenylate cyclase 9 | NM_001116.3 | 7 |
| CS | Homo sapiens citrate synthase | NM_004077.2 | 7 |
| PIGT | Homo sapiens mRNA for phosphatidylinositol glycan, class T variant | NM_001184728.2 | 7 |
| RANBP2 | Homo sapiens RAN binding protein 2 (RANBP2), mRNA | NM_006267.4 | 7 |
| RFXANK | Homo sapiens regulatory factor X-associated ankyrin-containing protein, transcript variant 1 | NM_001278727.1 | 7 |
| ALOX5 | Homo sapiens mRNA for arachidonate 5-lipoxygenase variant protein | NM_000698.3 | 6 |
| RBMS2 | Homo sapiens RNA binding motif, single stranded interacting protein 2, mRNA | NM_002898.3 | 6 |
| HNRNPL | Homo sapiens heterogeneous nuclear ribonucleoprotein L (HNRNPL), transcript variant 1, mRNA | NM_001533.2 | 5 |
| COL12A1 | Homo sapiens collagen, type XII, alpha 1 (COL12A1), transcript variant long, mRNA | NM_004370.5 | 5 |
| PCBP1 | H.sapiens hnRNP-E1 mRNA | NM_006196.3 | 1 |
| **CDKN1B** | Homo sapiens cyclin-dependent kinase inhibitor 1B (p27, Kip1), mRNA | NM_004064.4 | 1 |
| **CDKN1A** | Homo.sapiens cyclin-dependent kinase inhibitor 1A (p21, Cip1), mRNA | NM_000389.4 | 1 |
| MYC | Homo sapiens v-myc myelocytomatosis viral oncogene homolog (avian), mRNA | NM_002467.4 | 1 |
| P4HA1 | Homo sapiens prolyl 4-hydroxylase, alpha polypeptide I, mRNA | NM_000917.3 | 1 |
| **NEFH** | Homo sapiens neurofilament, heavy polypeptide, mRNA | NM_021076.3 | 1 |
| **EIF4E1B** | Homo sapiens eukaryotic translation initiation factor 4E family member 1B (EIF4E1B), mRNA | NM_001099408.1 | 1 |
| HNRNPA0 | Homo sapiens heterogeneous nuclear ribonucleoprotein A0 (HNRNPA0), mRNA | NM_006805.3 | 1 |

| **Table S5 Correlation between clinicopathologic features with PCBP1 expression in renal cancer patients** | | | |
| --- | --- | --- | --- |
|  | **Variable** | **Case** | ***P* value** |
| **Age** | < 55  ≥ 55 | 128  288 | 0.744 |
| **Gender** | Female  Male | 148  268 | 0.570 |
| **Stage** | I  II  III  IV | 164  55  105  92 | 0.001 ** |
| **PCBP1** | Low (<110 FPKM)  High (≥110 FPKM) | 213  203 |  |
