# Supplementary material for: PCBP1 depletion promotes tumorigenesis through attenuation of p27Kip1 mRNA stability and translation
Source: J Exp Clin Cancer Res. 2018 Aug 7;37:187. doi: 10.1186/s13046-018-0840-1 (PMC6081911; doi:10.1186/s13046-018-0840-1)
Supplement: Supplementary file 2 — Supplementary Experimental Procedures. (DOCX 19 kb) [file 13046_2018_840_MOESM2_ESM.docx]

**Supplementary Experimental Procedures**

**PCBP2 siRNAs and transfection**

Two PCBP2 specific siRNA 5’-GGCCUAUACCAUUCAAGGAtt-3’ (siRNA1) (Ghosh et al., 2008) and 5’-GGAUCUACUGAUAGGCAGGtt-3’ (siRNA2) (Waggoner et al., 2009) were synthesized by RiboBio and annealed. The siRNAs were transfected into GFP or PCBP1-overexpressing A2780 cells with X tremeGENE HP DNA transfect reagent (Roche). After 48 hours, cells were harvested and lyzed for RNA extraction (qRT-PCR) or p27 protein analysis.

**EdU staining**

For EdU staining, cells were cultured 12 h with basic medium containing 25 μM EdU, and then fixed with 4% paraformaldehyde-PBS for 30 min and treated with 0.1% TritonX-100 for 15 min. Then, cells incubated with Apollo reaction solution and Hoechst using Cell-Light^TM^ EdU Apollo®643 In Vitro Flow Cytometry Kit (Ribo Bio, C10338-2) followed by the manufacturer’s instruction. The treated cells were analyzed by flow cytometry (BD Biosciences or Beckman Gallios), or photographed using fluorescence microscope.

**Semi-quantitative RT-PCR detection of p27 expression in paired human colon tissues**

Total RNA was isolated from 10 paired colon tumor samples with Ultrapure RNA kit (CW Biotech) and the complementary DNA (cDNA) was synthesized by using Transcriptor First Strand cDNA Synthesis System kit (Roche) with Oligo (dT)_18_. For semi-quantitative analysis, PCR amplification was performed with GoTaq^®^ DNA polymerase (Promega), 1 μM of each pair of the indicated primers (Table S1), and 0.5 μl cDNA for a 2 min initial denaturation at 95 ^o^C, followed by 20-27 cycles of 30 s at 95 ^o^C, 30 s at the appropriate anneal time, and 1 min at 72 ^o^C. Products were run in 1.5 % Agarose gel and the band intensity was scanned and normalized based on their corresponding internal GAPDH control as the relative expression level.

**Renal tumor dataset analysis**

# Dataset containing 877 renal tumor samples were collected from The Human Protein Atlas database (<https://www.proteinatlas.org/ENSG00000169564-PCBP1/pathology/tissue/renal+cancer>), and the incomplete data without following up or less than 5 years after the first diagnosis were excluded. Bivariate correlations between PCBP1 with clinical parameters and the survival curve of 416 Samples of dead patients and live patients who observed more than five years were analyzed by using SPSS13.0. The statistical significance was defined as p<0.05.

# Supplemental references:

Ghosh D, Srivastava GP, Xu D, Schulz LC, Roberts RM. A link between SIN1 (MAPKAP1) and poly(rC) binding protein 2 (PCBP2) in counteracting environmental stress. Proc Natl Acad Sci U S A. 2008, 105 (33): 11673-8.

Waggoner, S, A.Johannes, G, J.Liebhaber, S. A. Depletion of the poly(C)-binding proteins alphaCP1 and alphaCP2 from K562 cells leads to p53-independent induction of cyclin-dependent kinase inhibitor (CDKN1A) and G1 arrest. J Biol Chem. 2009, 284(14): 9039-49.
