# Supplementary material for: PCBP1 depletion promotes tumorigenesis through attenuation of p27Kip1 mRNA stability and translation
Source: J Exp Clin Cancer Res. 2018 Aug 7;37:187. doi: 10.1186/s13046-018-0840-1 (PMC6081911; doi:10.1186/s13046-018-0840-1)
Supplement: Supplementary file 3 — Figure S1. PCBP1 increased p27 mRNA stability in A2780, DLD-1 and MDA-MB-231 cells. (A). Schematic procedure of isolation and identification of PCBP1-bound RNA transcripts. (B). Semi-quantitative RT-PCR detection of p27 mRNA levels in the indicated cell lines. A positive correlation between PCBP1 and p27 mRNA level is observed in these cell lines. (PPT 740 kb) [file 13046_2018_840_MOESM3_ESM.ppt]

## Slide 1
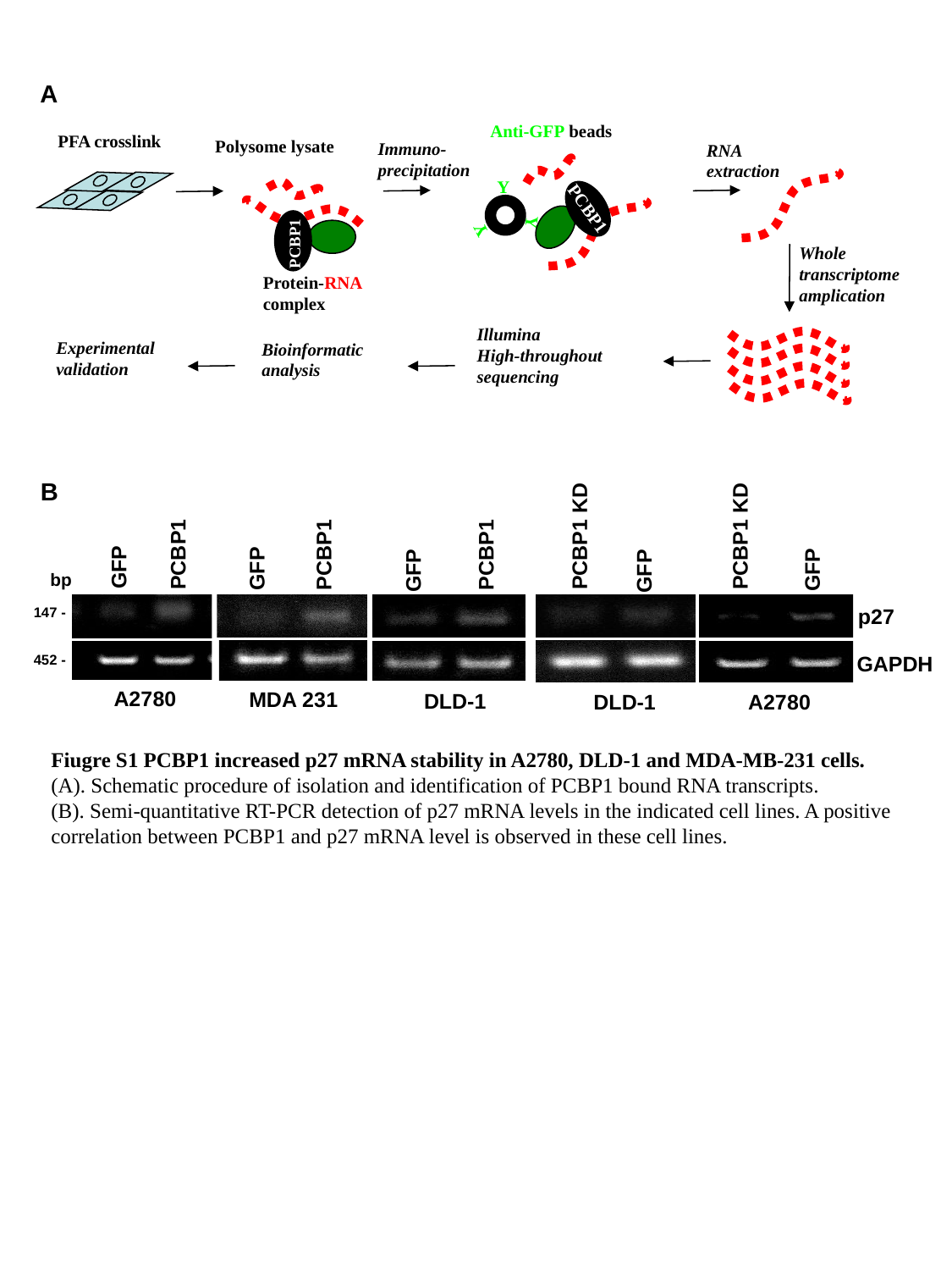

A
Anti-GFP beads
PFA crosslink
Polysome lysate
Immuno-precipitation
RNA
extraction
Y
Y
PCBP1
Y
PCBP1
Whole transcriptome amplication
Protein-RNA complex
Illumina
High-throughout
sequencing
Experimental
validation
Bioinformatic
analysis
B
PCBP1 KD
PCBP1 KD
PCBP1
PCBP1
PCBP1
GFP
GFP
GFP
GFP
GFP
p27
GAPDH
A2780
MDA 231
DLD-1
DLD-1
A2780
bp
147 ­-
452 - ­
Fiugre S1 PCBP1 increased p27 mRNA stability in A2780, DLD-1 and MDA-MB-231 cells.
(A). Schematic procedure of isolation and identification of PCBP1 bound RNA transcripts.
(B). Semi-quantitative RT-PCR detection of p27 mRNA levels in the indicated cell lines. A positive correlation between PCBP1 and p27 mRNA level is observed in these cell lines.
