# Supplementary figures and images for: PCBP1 depletion promotes tumorigenesis through attenuation of p27Kip1 mRNA stability and translation
Source: J Exp Clin Cancer Res. 2018 Aug 7;37:187. doi: 10.1186/s13046-018-0840-1 (PMC6081911; doi:10.1186/s13046-018-0840-1)

## Slide 1
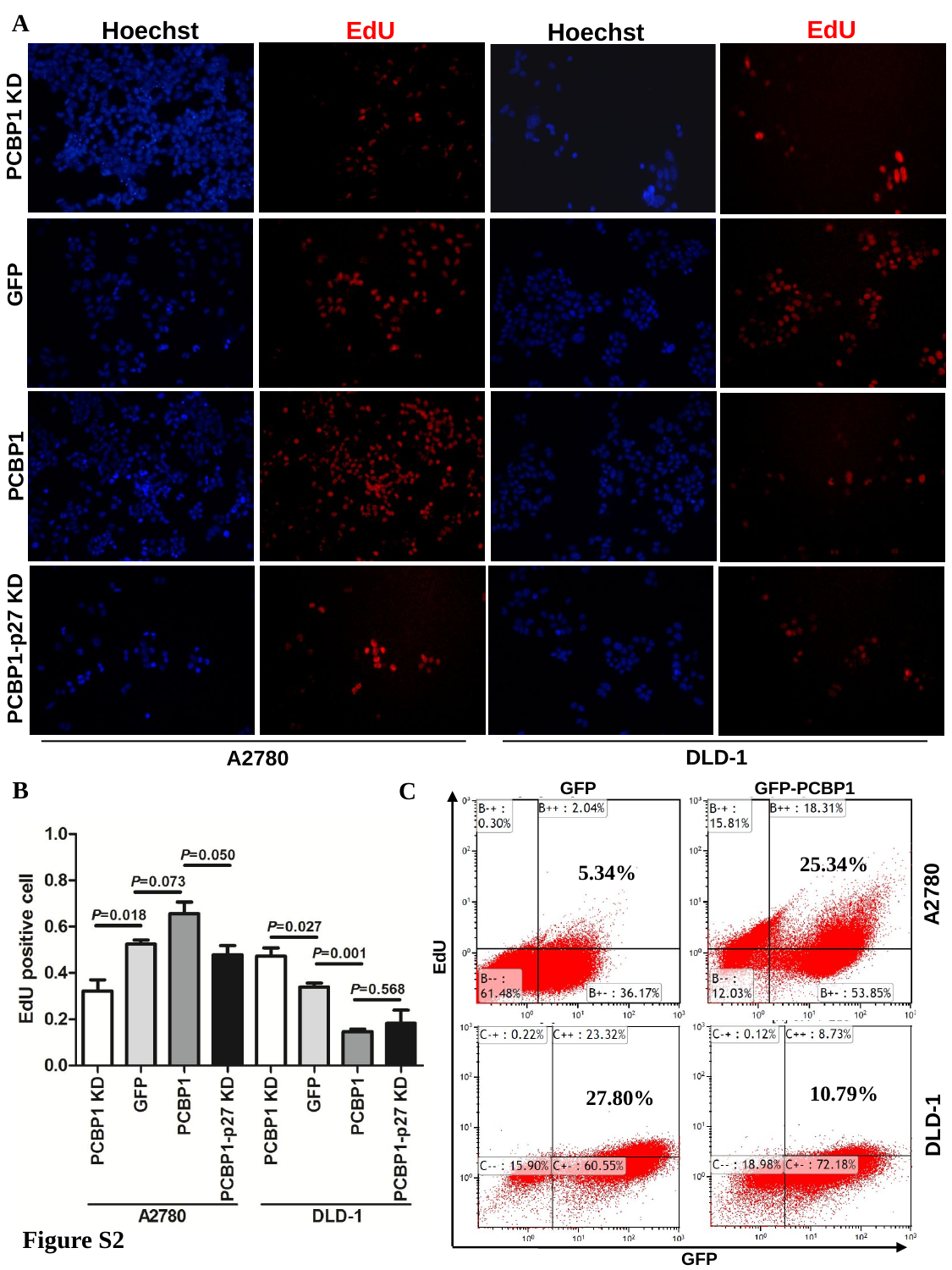

A
EdU
Hoechst
EdU
Hoechst
PCBP1 KD
GFP
PCBP1
PCBP1-p27 KD
DLD-1
A2780
B
C
GFP
GFP-PCBP1
25.34%
5.34%
A2780
EdU
10.79%
27.80%
DLD-1
GFP
Figure S2

Supplement: Supplementary file 4 — Figure S2. PCBP1 overexpression inhibits cell cycle progression. (A). Representative immunofluorescence staining of A2780 and DLD-1 cells with EdU and Hoechst in cells with endogenous PCBP1 knockdown (PCBP1 KD), GFP-PCBP1 overexpression (PCBP1) and the additional p27KD in PCBP1-overexpressing cells (PCBP1-p27KD). (B). Statistical analyses of EdU positive cells in A. The EdU positive cells were counted randomly in 3 views and analyzed. (mean ± SEM, n = 3 per group). (C). Representative flow cytometry analysis of A2780 and DLD-1 cells with PCBP1 overexpression after EDU staining. Ratios of EdU + GFP-positive cells to GFP positive cells or GFP-PCBP1-positive cells are shown in the right-upper quadrant of each diagram. (PPT 2149 kb) [file 13046_2018_840_MOESM4_ESM.ppt]
