# Supplementary material for: PCBP1 depletion promotes tumorigenesis through attenuation of p27Kip1 mRNA stability and translation
Source: J Exp Clin Cancer Res. 2018 Aug 7;37:187. doi: 10.1186/s13046-018-0840-1 (PMC6081911; doi:10.1186/s13046-018-0840-1)
Supplement: Supplementary file 5 — Figure S3. Knockdown efficiency of p27 in A2780 and DLD-1 cells. (A). Immunoblots of p27 in DLD-1 and A2780 cells upon its two specific shRNAs transfection after 46 h. (B) Immunoblots of p27 in the stably PCBP1-overexpressing DLD-1 and A2780 cells with supplemental p27 KD with two specific shRNAs. (PPT 2285 kb) [file 13046_2018_840_MOESM5_ESM.ppt]

## Slide 1
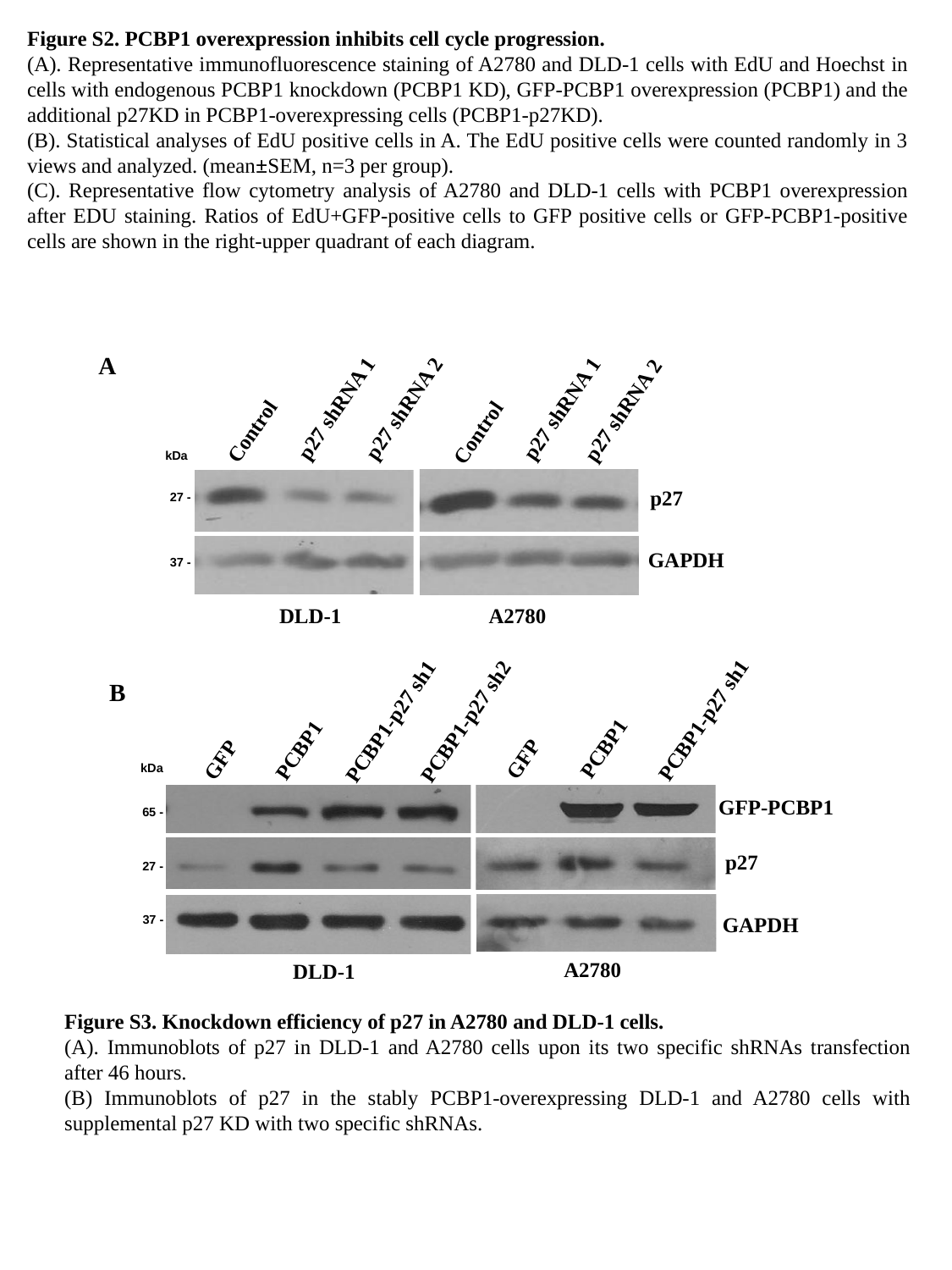

Figure S2. PCBP1 overexpression inhibits cell cycle progression.
(A). Representative immunofluorescence staining of A2780 and DLD-1 cells with EdU and Hoechst in cells with endogenous PCBP1 knockdown (PCBP1 KD), GFP-PCBP1 overexpression (PCBP1) and the additional p27KD in PCBP1-overexpressing cells (PCBP1-p27KD).
(B). Statistical analyses of EdU positive cells in A. The EdU positive cells were counted randomly in 3 views and analyzed. (mean±SEM, n=3 per group).
(C). Representative flow cytometry analysis of A2780 and DLD-1 cells with PCBP1 overexpression after EDU staining. Ratios of EdU+GFP-positive cells to GFP positive cells or GFP-PCBP1-positive cells are shown in the right-upper quadrant of each diagram.
p27 shRNA 2
p27 shRNA 1
p27 shRNA 2
p27 shRNA 1
Control
Control
p27
GAPDH
DLD-1
A2780
kDa
27 - ­
37 -­
A
PCBP1
PCBP1
GFP
GFP
GFP-PCBP1
p27
GAPDH
A2780
DLD-1
PCBP1-p27 sh2
PCBP1-p27 sh1
PCBP1-p27 sh1
kDa
65 -­
27 -­
37 - ­
B
Figure S3. Knockdown efficiency of p27 in A2780 and DLD-1 cells.
(A). Immunoblots of p27 in DLD-1 and A2780 cells upon its two specific shRNAs transfection after 46 hours.
(B) Immunoblots of p27 in the stably PCBP1-overexpressing DLD-1 and A2780 cells with supplemental p27 KD with two specific shRNAs.
