# Supplementary material for: PCBP1 depletion promotes tumorigenesis through attenuation of p27Kip1 mRNA stability and translation
Source: J Exp Clin Cancer Res. 2018 Aug 7;37:187. doi: 10.1186/s13046-018-0840-1 (PMC6081911; doi:10.1186/s13046-018-0840-1)
Supplement: Supplementary file 6 — Figure S4. Colonies induced by A2780 or DLD-1 cells overexpressing PCBP1. PCBP1, PCBP1-p27 KD and GFP control cells were analyzed by soft agar assay for anchorage-independent growth. Colonies were stained with Trypan Blue and photographed. (PPT 609 kb) [file 13046_2018_840_MOESM6_ESM.ppt]

## Slide 1
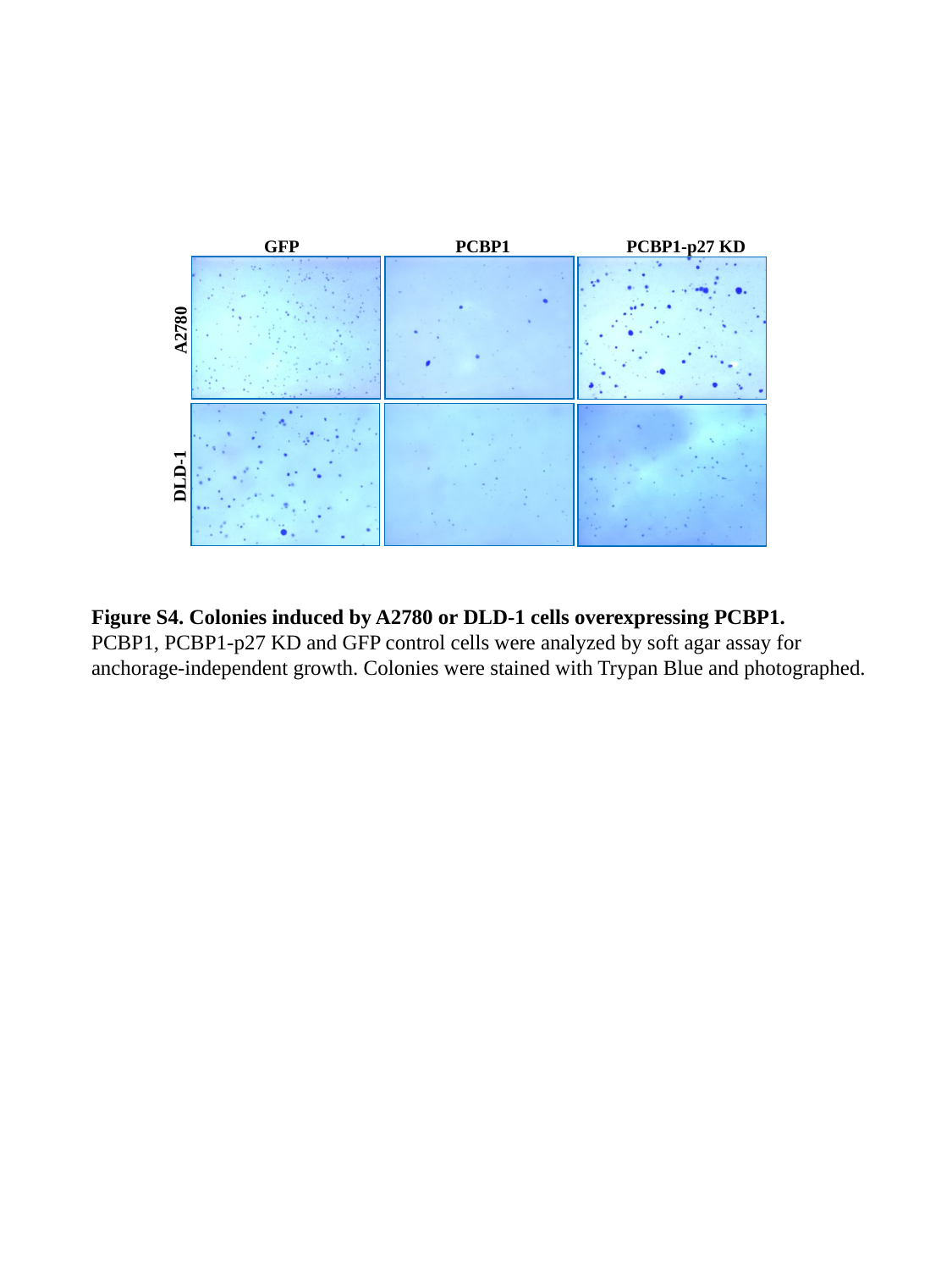

GFP
PCBP1
PCBP1-p27 KD
A2780
DLD-1
Figure S4. Colonies induced by A2780 or DLD-1 cells overexpressing PCBP1.
PCBP1, PCBP1-p27 KD and GFP control cells were analyzed by soft agar assay for anchorage-independent growth. Colonies were stained with Trypan Blue and photographed.
