# Supplementary material for: PCBP1 depletion promotes tumorigenesis through attenuation of p27Kip1 mRNA stability and translation
Source: J Exp Clin Cancer Res. 2018 Aug 7;37:187. doi: 10.1186/s13046-018-0840-1 (PMC6081911; doi:10.1186/s13046-018-0840-1)
Supplement: Supplementary file 7 — Figure S5. p27 mRNA stabilized by PCBP1. The half-life of p27 mRNA was derived from the decay curve plotted from the Fig. 6g and h. Linear regression equation in A: PCBP1, y = − 0.0096× + 0.998,R2 = 0.99; GFP, y = − 0.058× + 0.9844, R2 = 0.9865; B: PCBP1 KD, y = − 0.0749× + 0.965, R2 = 0.9607; GFP, y = − 0.0749× + 0.9677, R2 = 0.9387. (PPT 309 kb) [file 13046_2018_840_MOESM7_ESM.ppt]

## Slide 1
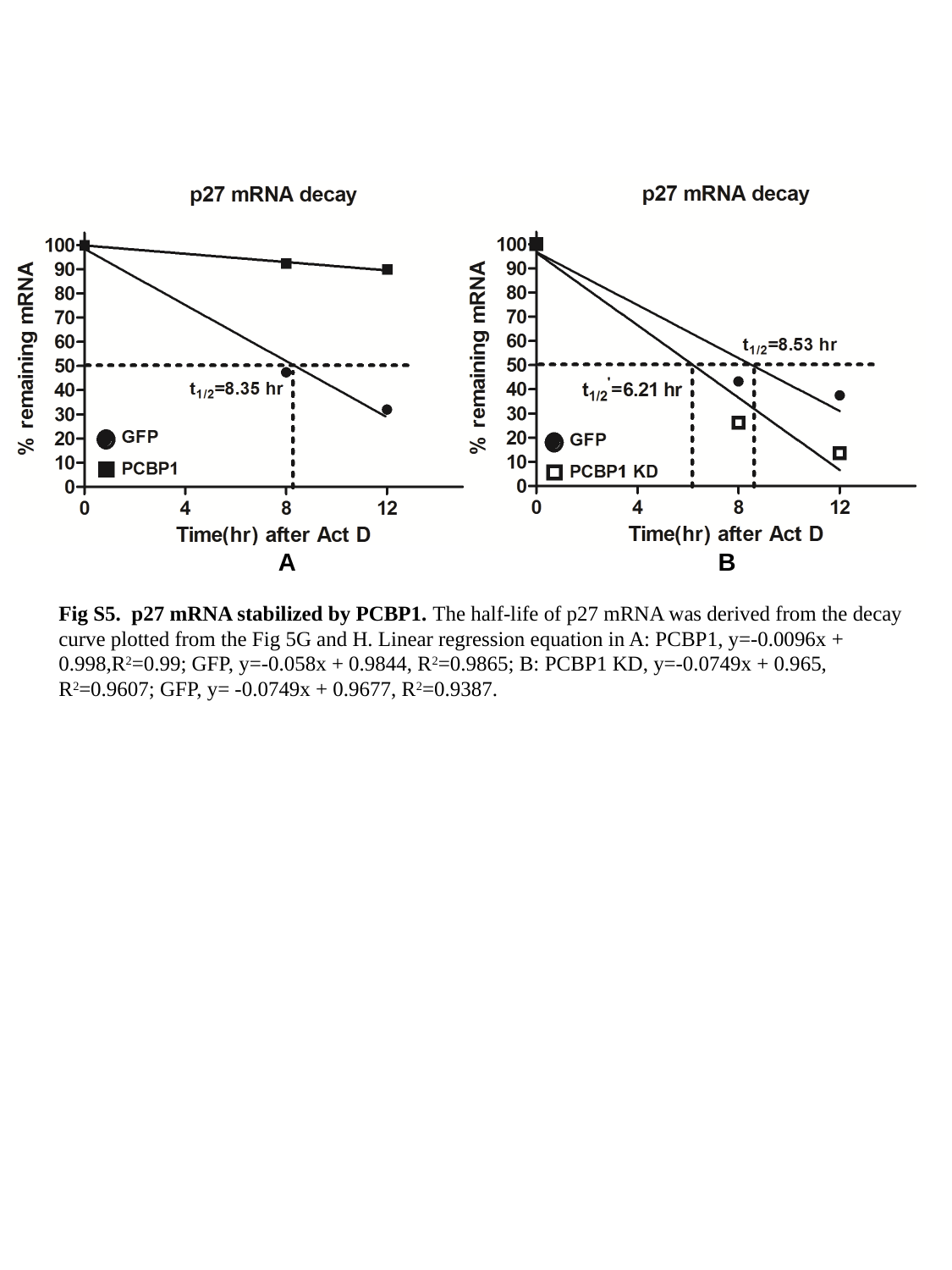

A
B
Fig S5. p27 mRNA stabilized by PCBP1. The half-life of p27 mRNA was derived from the decay curve plotted from the Fig 5G and H. Linear regression equation in A: PCBP1, y=-0.0096x + 0.998,R2=0.99; GFP, y=-0.058x + 0.9844, R2=0.9865; B: PCBP1 KD, y=-0.0749x + 0.965, R2=0.9607; GFP, y= -0.0749x + 0.9677, R2=0.9387.
