# Supplementary material for: PCBP1 depletion promotes tumorigenesis through attenuation of p27Kip1 mRNA stability and translation
Source: J Exp Clin Cancer Res. 2018 Aug 7;37:187. doi: 10.1186/s13046-018-0840-1 (PMC6081911; doi:10.1186/s13046-018-0840-1)
Supplement: Supplementary file 8 — Figure S6. No effect of PCBP2 on p27 expression on both mRNA and protein levels. (A). RT-PCR analysis of PCBP2 knockdown efficiency by 2 specific siRNAs in A2780 GFP control cells or GFP-PCBP1 overexpressing cells. (B). Immunoblot analysis of p27 expression upon PCBP2 knockdown in A2780 GFP control cells or GFP-PCBP1 overexpressing cells. GAPDH was used as a loading control. (PPT 2665 kb) [file 13046_2018_840_MOESM8_ESM.ppt]

## Slide 1
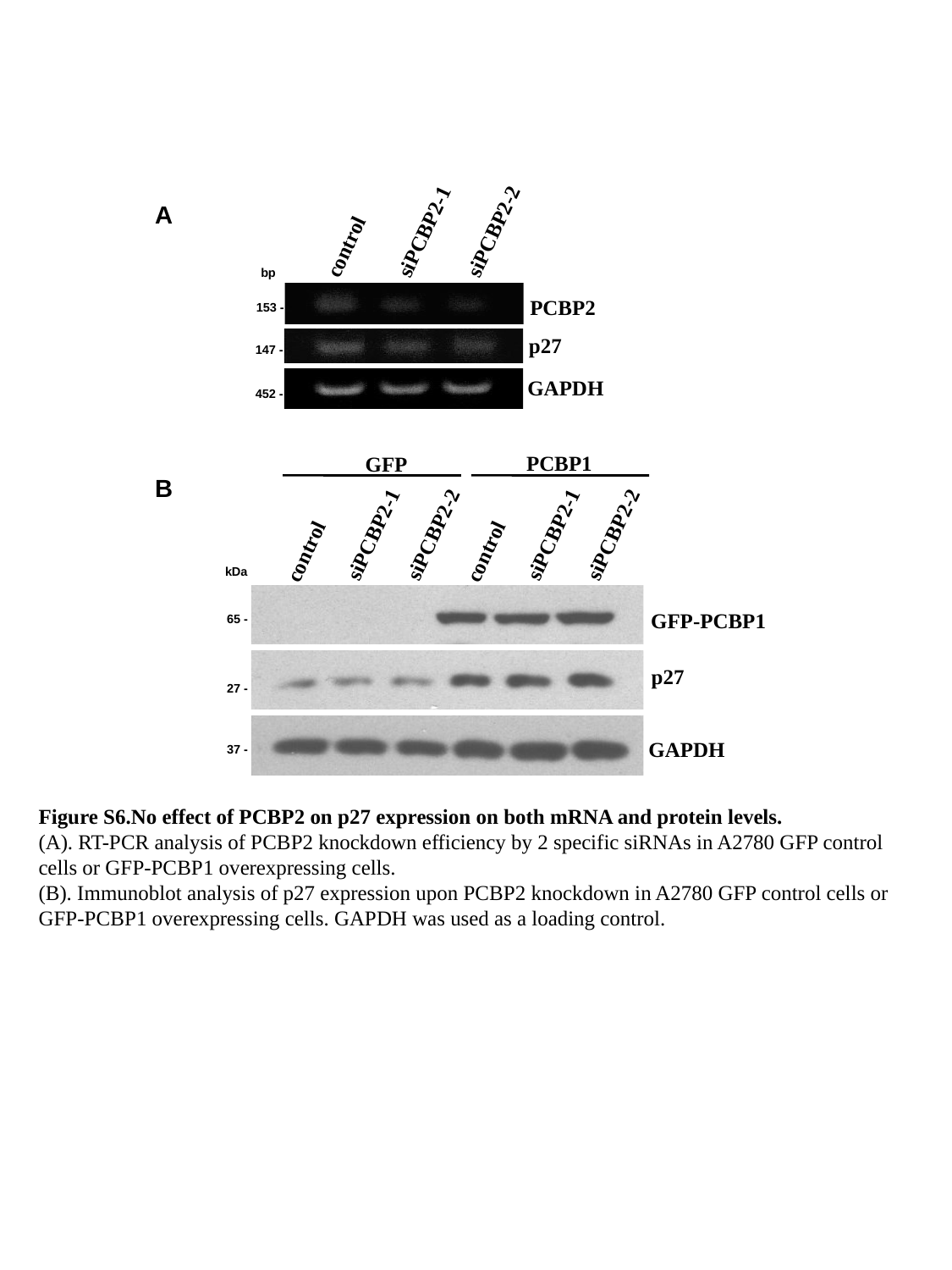

siPCBP2-2
siPCBP2-1
control
PCBP2
p27
GAPDH
bp
153 -­
147 -­
452 ­-
A
PCBP1
GFP
siPCBP2-1
siPCBP2-2
siPCBP2-1
siPCBP2-2
control
control
GFP-PCBP1
p27
GAPDH
kDa
65 -­
27 -­
37­ -
B
Figure S6.No effect of PCBP2 on p27 expression on both mRNA and protein levels.
(A). RT-PCR analysis of PCBP2 knockdown efficiency by 2 specific siRNAs in A2780 GFP control cells or GFP-PCBP1 overexpressing cells.
(B). Immunoblot analysis of p27 expression upon PCBP2 knockdown in A2780 GFP control cells or GFP-PCBP1 overexpressing cells. GAPDH was used as a loading control.
