# Supplementary material for: PCBP1 depletion promotes tumorigenesis through attenuation of p27Kip1 mRNA stability and translation
Source: J Exp Clin Cancer Res. 2018 Aug 7;37:187. doi: 10.1186/s13046-018-0840-1 (PMC6081911; doi:10.1186/s13046-018-0840-1)
Supplement: Supplementary file 9 — Figure S7. Expression of PCBP1 and p27 in ovary cancer samples compared to that in the normal tissues. The indicated protein expression level was defined based on the staining intensity under the same robust IHC staining condition. (PPT 1277 kb) [file 13046_2018_840_MOESM9_ESM.ppt]

## Slide 1
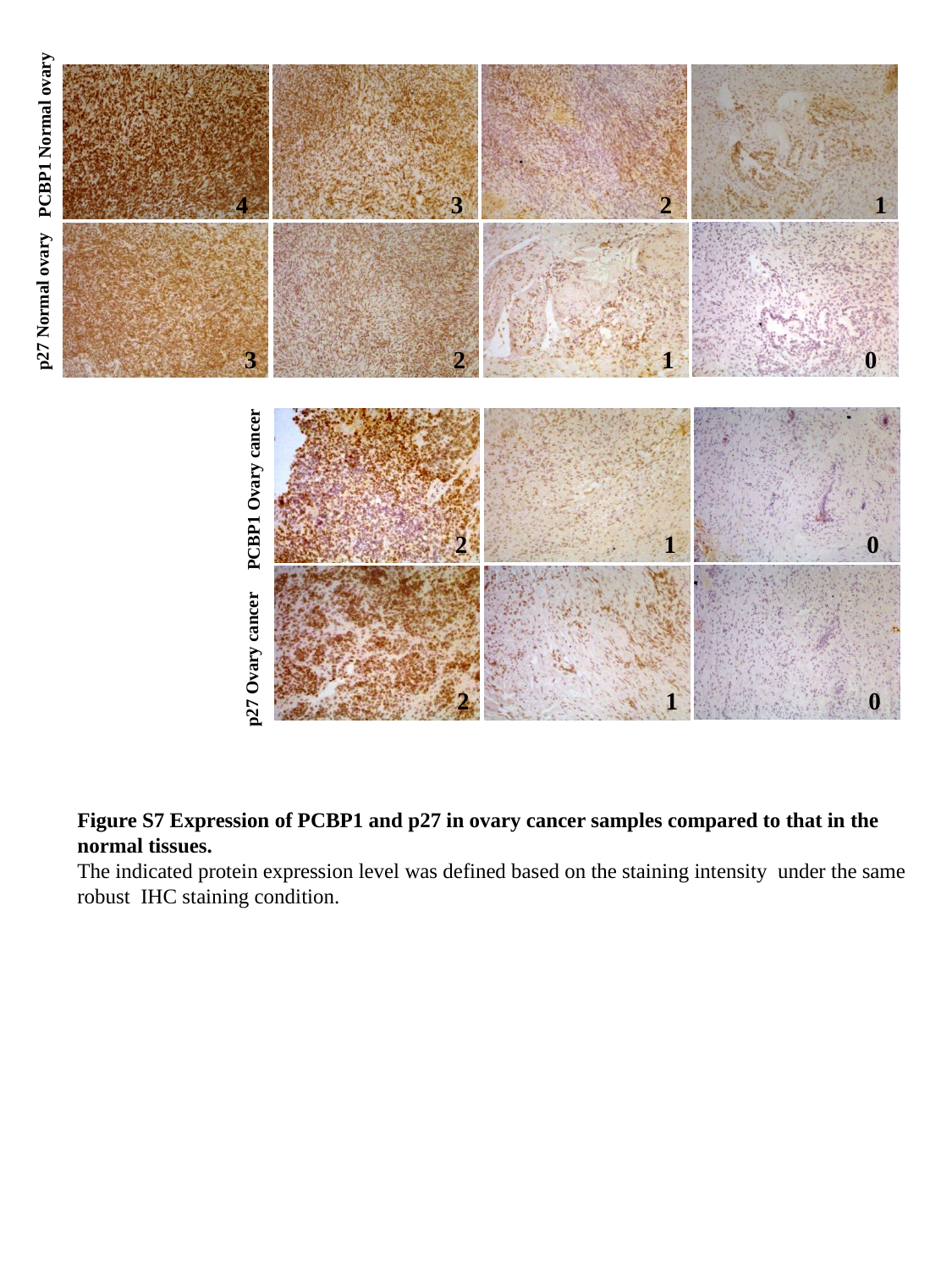

PCBP1 Normal ovary
4 3 2 1
p27 Normal ovary
3 2 1 0
PCBP1 Ovary cancer
 2 1 0
p27 Ovary cancer
 2 1 0
Figure S7 Expression of PCBP1 and p27 in ovary cancer samples compared to that in the normal tissues.
The indicated protein expression level was defined based on the staining intensity under the same robust IHC staining condition.
