# Supplementary material for: PCBP1 depletion promotes tumorigenesis through attenuation of p27Kip1 mRNA stability and translation
Source: J Exp Clin Cancer Res. 2018 Aug 7;37:187. doi: 10.1186/s13046-018-0840-1 (PMC6081911; doi:10.1186/s13046-018-0840-1)
Supplement: Supplementary file 10 — Figure S8. Expression of PCBP1 and p27 in paired colon cancer samples compared to that in the normal tissues. The indicated protein expression level was defined based on the staining intensity under the same robust IHC staining condition. (PPT 1072 kb) [file 13046_2018_840_MOESM10_ESM.ppt]

## Slide 1
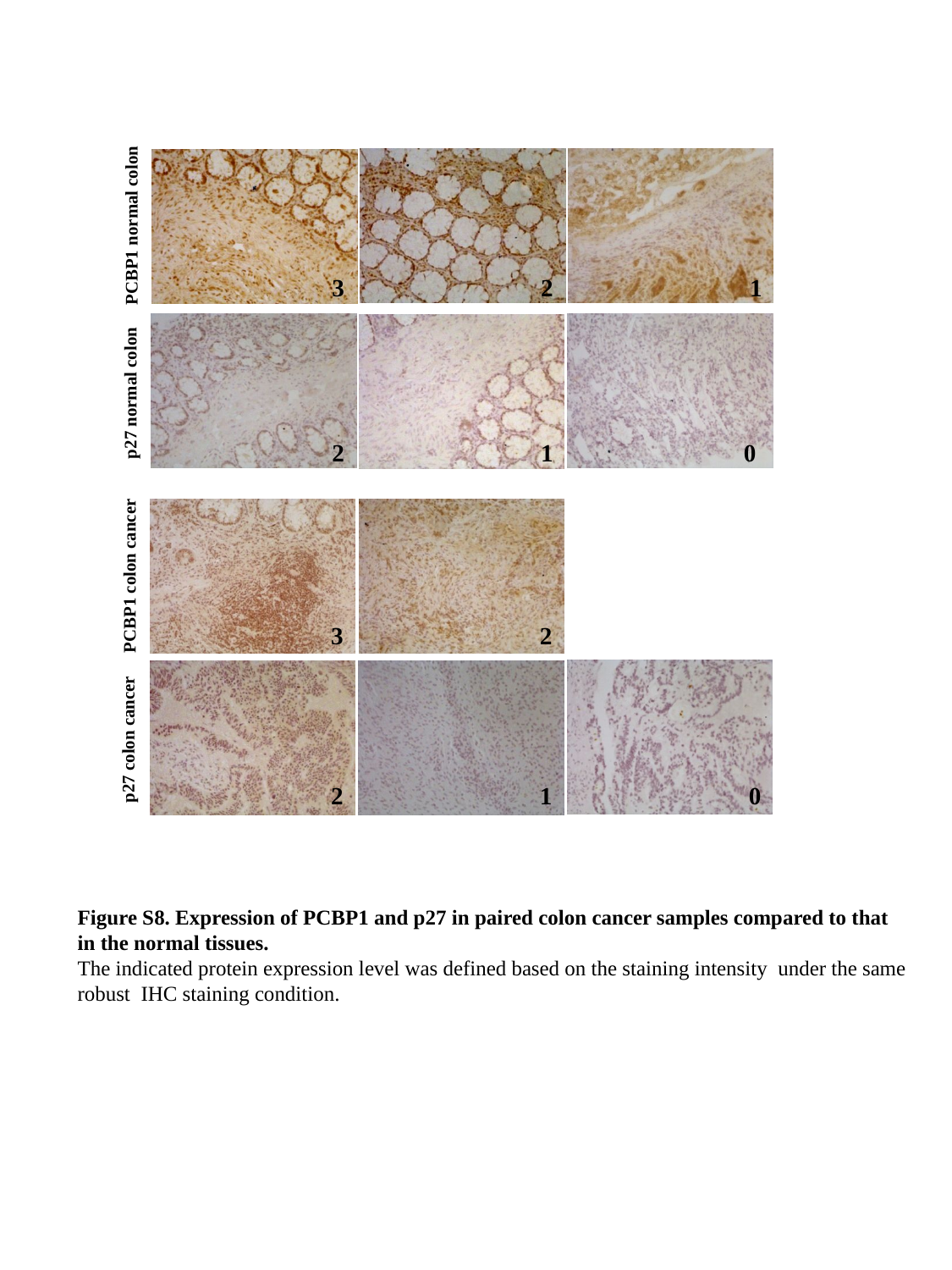

PCBP1 normal colon
3 2 1
p27 normal colon
2 1 0
PCBP1 colon cancer
3 2
p27 colon cancer
2 1 0
Figure S8. Expression of PCBP1 and p27 in paired colon cancer samples compared to that in the normal tissues.
The indicated protein expression level was defined based on the staining intensity under the same robust IHC staining condition.
