# Supplementary material for: PCBP1 depletion promotes tumorigenesis through attenuation of p27Kip1 mRNA stability and translation
Source: J Exp Clin Cancer Res. 2018 Aug 7;37:187. doi: 10.1186/s13046-018-0840-1 (PMC6081911; doi:10.1186/s13046-018-0840-1)
Supplement: Supplementary file 11 — Figure S9. Relationship of PCBP1 to p27 mRNA level in tumor samples. (A). Semiquantitative RT-PCR detection of PCBP1, p27 mRNA expression in conlon tumor tissues. GAPDH was used as control. p27 mRNA level is correlated to PCBP1 level in the paired normal (N) and tumor (T) samples of colon tissues. (B). p27 mRNA level as well as its ubiquitin ligase Skp2 in ovarian epithelia. GEO ID is shown. (C). TCGA gene profile of Skp2 in tumors indicates the relative low amplification in ovary and colon tumors. (PPT 2633 kb) [file 13046_2018_840_MOESM11_ESM.ppt]

## Slide 1
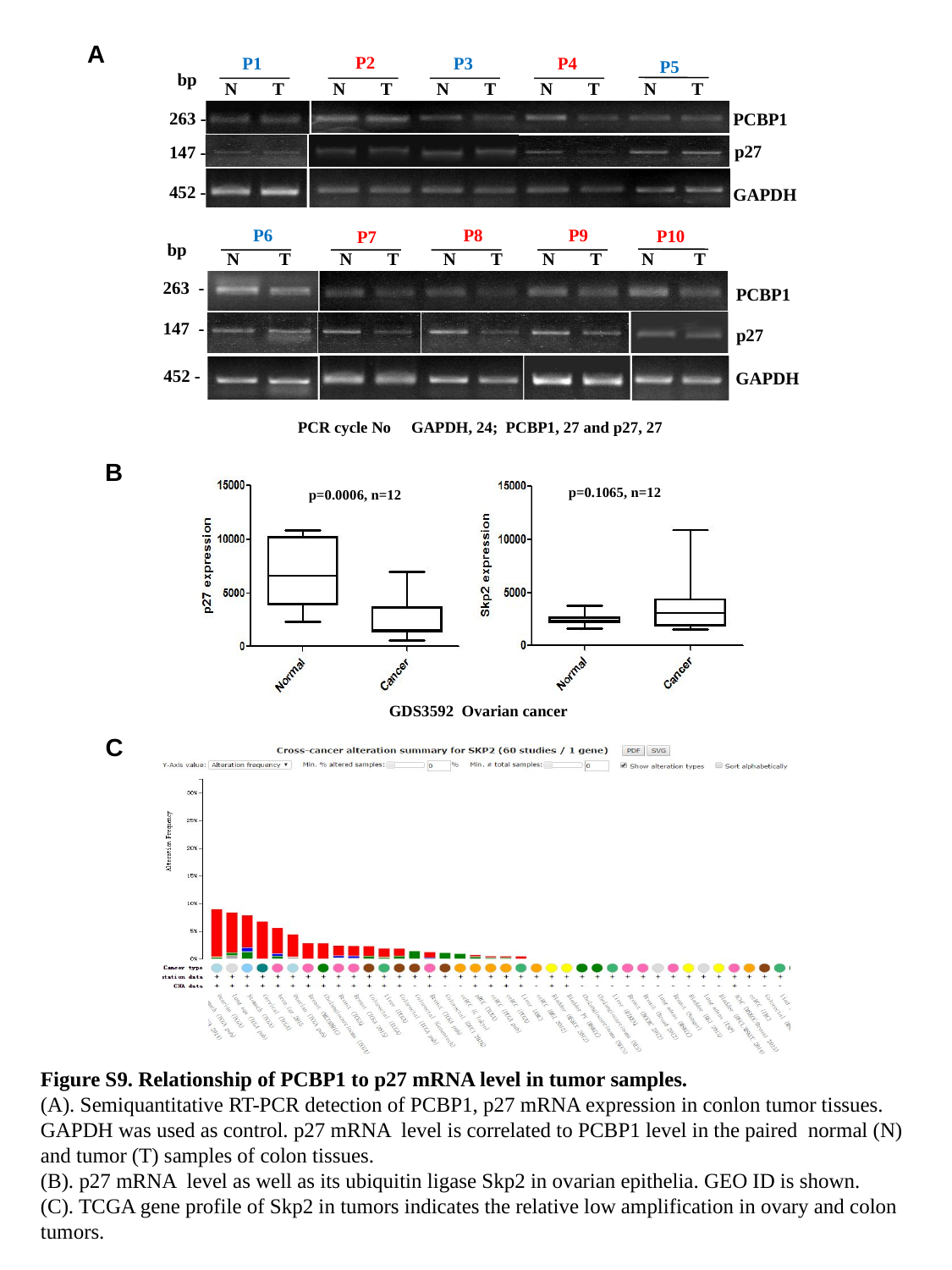

A
P2
P1
P3
P4
P5
bp
 N T N T N T N T N T
263 - ­
PCBP1
p27
147­ -
452 -­
GAPDH
P6
P8
P9
P10
P7
bp
 N T N T N T N T N T
263 -­
PCBP1
147­ -
p27
452­ -
GAPDH
PCR cycle No：GAPDH, 24; PCBP1, 27 and p27, 27
B
p=0.0006, n=12
p=0.1065, n=12
GDS3592 Ovarian cancer
C
Figure S9. Relationship of PCBP1 to p27 mRNA level in tumor samples.
(A). Semiquantitative RT-PCR detection of PCBP1, p27 mRNA expression in conlon tumor tissues. GAPDH was used as control. p27 mRNA level is correlated to PCBP1 level in the paired normal (N) and tumor (T) samples of colon tissues.
(B). p27 mRNA level as well as its ubiquitin ligase Skp2 in ovarian epithelia. GEO ID is shown.
(C). TCGA gene profile of Skp2 in tumors indicates the relative low amplification in ovary and colon tumors.
