# Supplementary material for: PCBP1 depletion promotes tumorigenesis through attenuation of p27Kip1 mRNA stability and translation
Source: J Exp Clin Cancer Res. 2018 Aug 7;37:187. doi: 10.1186/s13046-018-0840-1 (PMC6081911; doi:10.1186/s13046-018-0840-1)
Supplement: Supplementary file 12 — Figure S10. A positive correlation between PCBP1 and p27 proteinlevels in human renal cancer samples. (B) Relationship between PCBP1 and p27 protein expression in IHC staining. A positive correlation between PCBP1 and p27 protein levels is observed in tumors from 7 patients. (C) RT-PCR detection of PCBP1, p27 mRNA exprission in renal tumor tissues. GAPDH was used as control. (D) Immunoblot of PCBP1 and p27 proteins in healthy tissues (N), tumor adjacent region (A) and tumor tissues (T). GAPDH was used as control. E. Kaplan-Meier survival curve of renal carcinoma patients with different expression level of PCBP1. Data is from https://www.proteinatlas.org/ENSG00000169564-PCBP1/pathology/tissue/renal+cancer. (PPT 2557 kb) [file 13046_2018_840_MOESM12_ESM.ppt]

## Slide 1
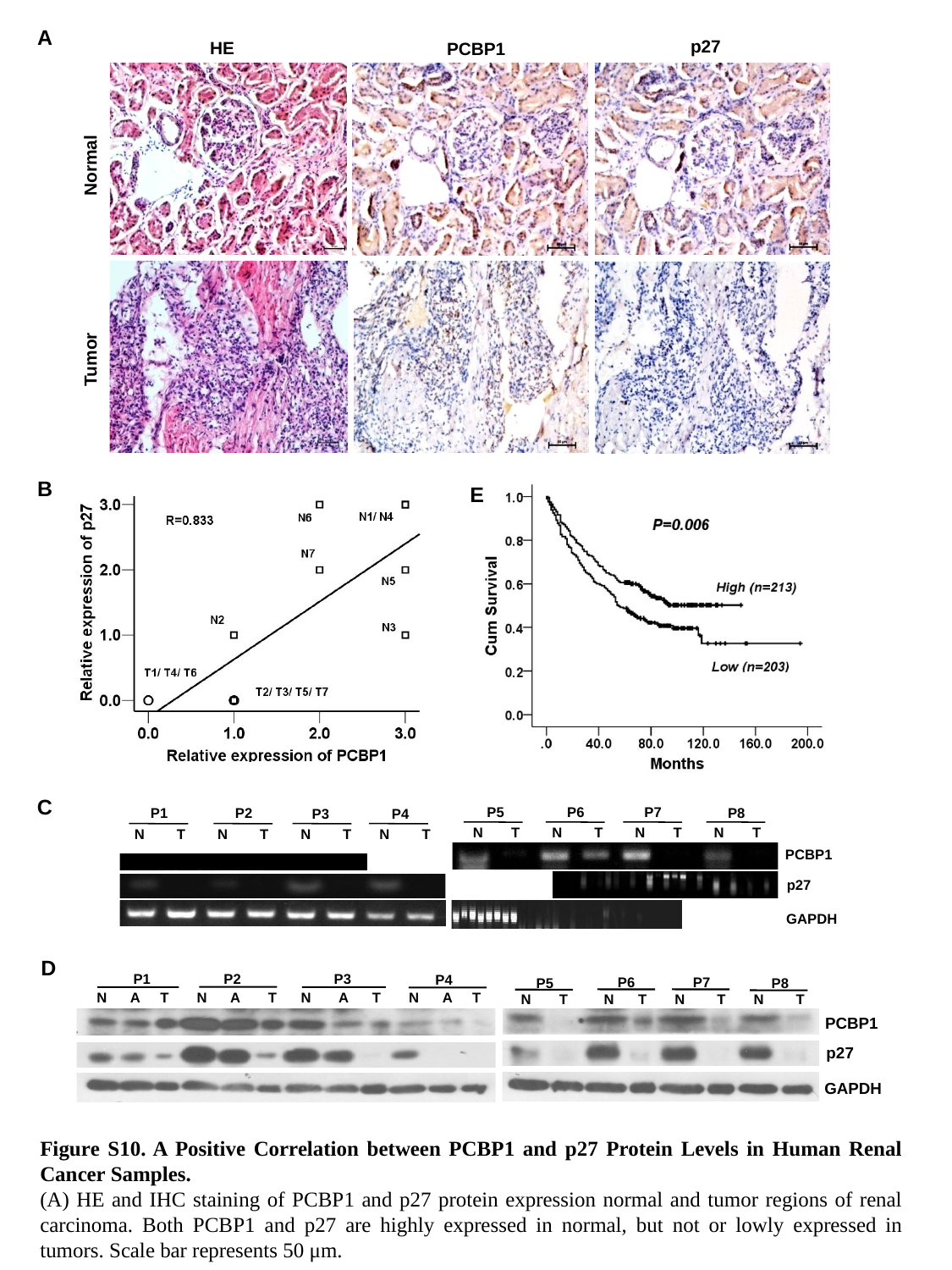

A
p27
HE
PCBP1
Normal
Tumor
B
E
C
P5
P6
P7
P1
P2
P8
P3
P4
N T N T N T N T
N T N T N T N T
PCBP1
p27
GAPDH
D
P2
 P1
P3
P4
N A T N A T N A T N A T
P6
P7
P5
P8
N T N T N T N T
PCBP1
p27
GAPDH
Figure S10. A Positive Correlation between PCBP1 and p27 Protein Levels in Human Renal Cancer Samples.
(A) HE and IHC staining of PCBP1 and p27 protein expression normal and tumor regions of renal carcinoma. Both PCBP1 and p27 are highly expressed in normal, but not or lowly expressed in tumors. Scale bar represents 50 μm.

## Slide 2
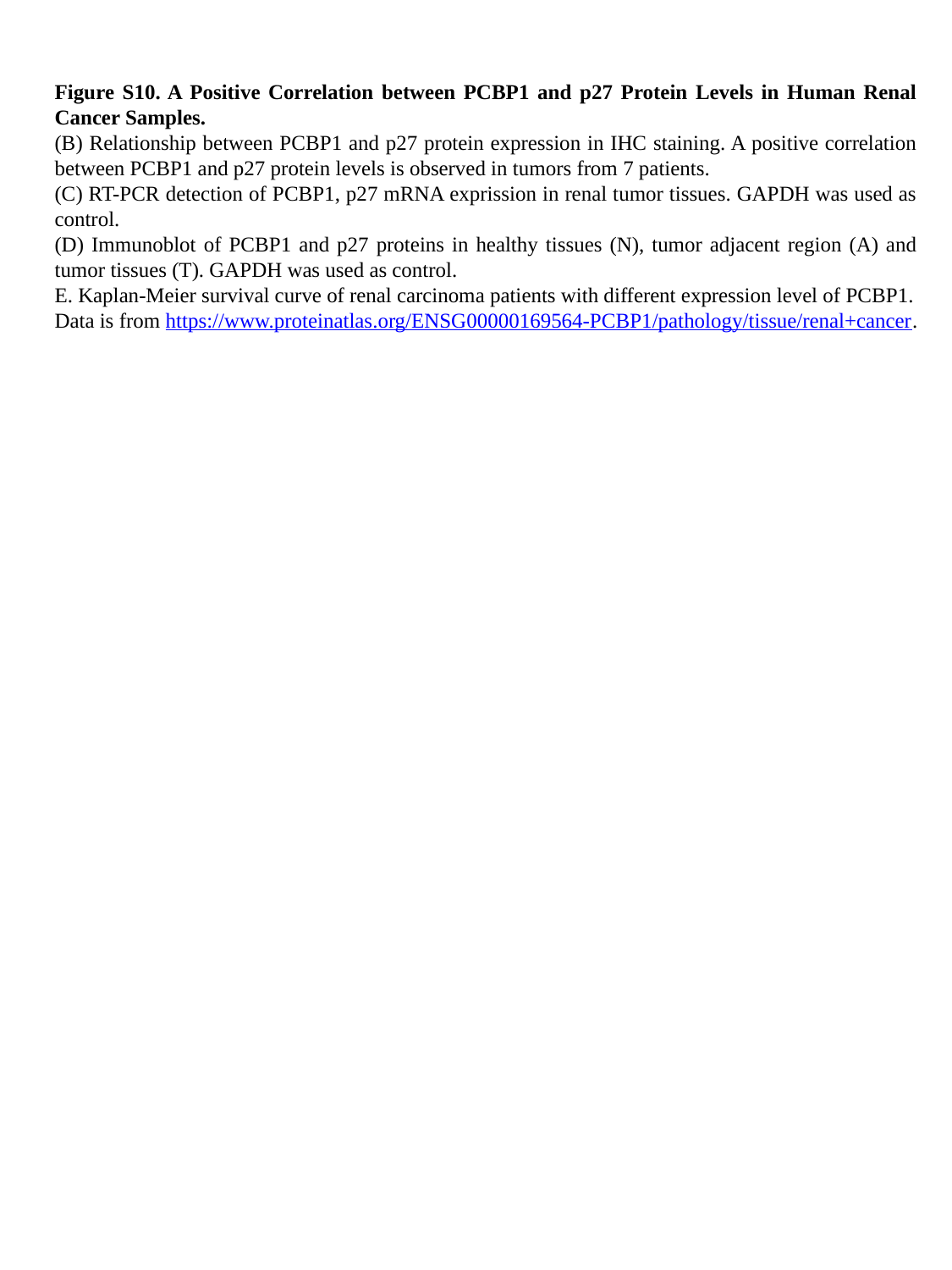

Figure S10. A Positive Correlation between PCBP1 and p27 Protein Levels in Human Renal Cancer Samples.
(B) Relationship between PCBP1 and p27 protein expression in IHC staining. A positive correlation between PCBP1 and p27 protein levels is observed in tumors from 7 patients.
(C) RT-PCR detection of PCBP1, p27 mRNA exprission in renal tumor tissues. GAPDH was used as control.
(D) Immunoblot of PCBP1 and p27 proteins in healthy tissues (N), tumor adjacent region (A) and tumor tissues (T). GAPDH was used as control.
E. Kaplan-Meier survival curve of renal carcinoma patients with different expression level of PCBP1.
Data is from https://www.proteinatlas.org/ENSG00000169564-PCBP1/pathology/tissue/renal+cancer.
