# Supplementary material for: PCBP1 depletion promotes tumorigenesis through attenuation of p27Kip1 mRNA stability and translation
Source: J Exp Clin Cancer Res. 2018 Aug 7;37:187. doi: 10.1186/s13046-018-0840-1 (PMC6081911; doi:10.1186/s13046-018-0840-1)
Supplement: Supplementary file 13 — Figure S11. PCBP1 and p27 expression in tumor cells are correlated to anti-cancer drug sensitivity. PCBP1 and p27 expression were analyzed by data from GEO database, and high expression of PCBP1 and p27 were observed in Tamoxifen (GSE26459), Doxorubicin (GSE24460) and Lapatinib (GSE16179) sensitive breast cancer subclones (n indicates the analyzed cell subclone numbers). (PPT 92 kb) [file 13046_2018_840_MOESM13_ESM.ppt]

## Slide 1
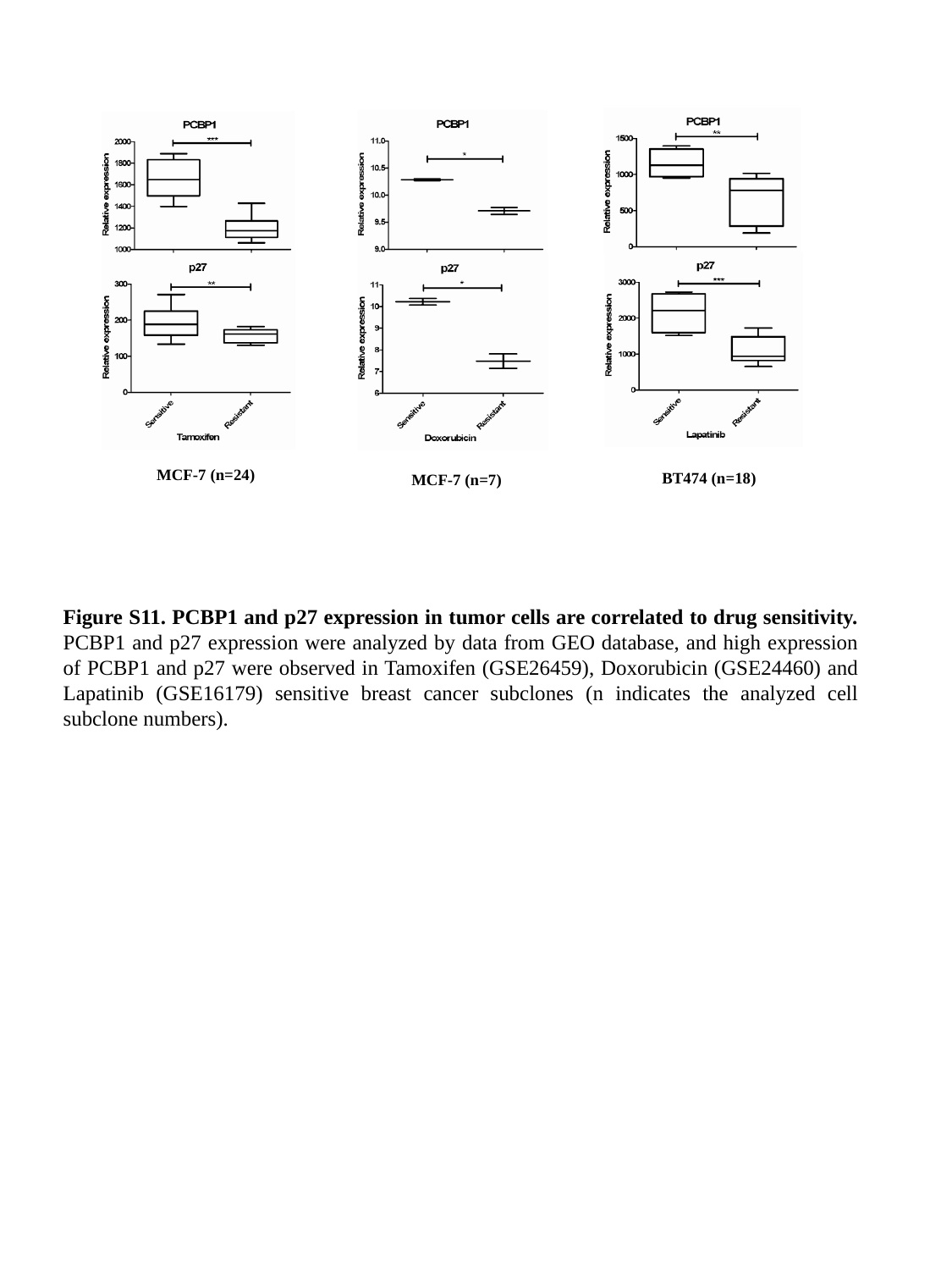

MCF-7 (n=24)
BT474 (n=18)
MCF-7 (n=7)
Figure S11. PCBP1 and p27 expression in tumor cells are correlated to drug sensitivity. PCBP1 and p27 expression were analyzed by data from GEO database, and high expression of PCBP1 and p27 were observed in Tamoxifen (GSE26459), Doxorubicin (GSE24460) and Lapatinib (GSE16179) sensitive breast cancer subclones (n indicates the analyzed cell subclone numbers).
